# Gender differentials and state variations in suicide deaths in India: the Global Burden of Disease Study 1990–2016

**DOI:** 10.1016/S2468-2667(18)30138-5

**Published:** 2018-09-12

**Authors:** India State-Level Disease Burden Initiative Suicide Collaborators, Rakhi Dandona, G Anil Kumar, R S Dhaliwal, Mohsen Naghavi, Theo Vos, D K Shukla, Lakshmi Vijayakumar, G Gururaj, J S Thakur, Atul Ambekar, Rajesh Sagar, Megha Arora, Deeksha Bhardwaj, Joy K Chakma, Eliza Dutta, Melissa Furtado, Scott Glenn, Caitlin Hawley, Sarah C Johnson, Tripti Khanna, Michael Kutz, W Cliff Mountjoy-Venning, Pallavi Muraleedharan, Thara Rangaswamy, Chris M Varghese, Mathew Varghese, K Srinath Reddy, Christopher J L Murray, Soumya Swaminathan, Lalit Dandona

## Abstract

**Background:**

A systematic understanding of suicide mortality trends over time at the subnational level for India's 1·3 billion people, 18% of the global population, is not readily available. Thus, we aimed to report time trends of suicide deaths, and the heterogeneity in its distribution between the states of India from 1990 to 2016.

**Methods:**

As part of the Global Burden of Diseases, Injuries, and Risk Factors Study (GBD) 2016, we estimated suicide death rates (SDRs) for both sexes in each state of India from 1990 to 2016. We used various data sources for estimating cause-specific mortality in India. For suicide mortality in India before 2000, estimates were based largely on GBD covariates. For each state, we calculated the ratio of the observed SDR to the rate expected in geographies globally with similar GBD Socio-demographic Index in 2016 (ie, the observed-to-expected ratio); and assessed the age distribution of suicide deaths, and the men-to-women ratio of SDR over time. Finally, we assessed the probability for India and the states of reaching the Sustainable Development Goal (SDG) target of a one-third reduction in SDR from 2015 to 2030, using location-wise trends of the age-standardised SDR from 1990 to 2016. We calculated 95% uncertainty intervals (UIs) for the point estimates.

**Findings:**

There were 230 314 (95% UI 194 058–250 260) suicide deaths in India in 2016. India's contribution to global suicide deaths increased from 25·3% in 1990 to 36·6% in 2016 among women, and from 18·7% to 24·3% among men. Age-standardised SDR among women in India reduced by 26·7% from 20·0 (95% UI 16·5–23·5) in 1990 to 14·7 (13·1–16·2) per 100 000 in 2016, but the age-standardised SDR among men was the same in 1990 (22·3 [95% UI 14·4–27·4] per 100 000) and 2016 (21·2 [14·6–23·6] per 100 000). SDR in women was 2·1 times higher in India than the global average in 2016, and the observed-to-expected ratio was 2·74, ranging from 0·45 to 4·54 between the states. SDR in men was 1·4 times higher in India than the global average in 2016, with an observed-to-expected ratio of 1·31, ranging from 0·40 to 2·42 between the states. There was a ten-fold variation between the states in the SDR for women and six-fold variation for men in 2016. The men-to-women ratio of SDR for India was 1·34 in 2016, ranging from 0·97 to 4·11 between the states. The highest age-specific SDRs among women in 2016 were for ages 15–29 years and 75 years or older, and among men for ages 75 years or older. Suicide was the leading cause of death in India in 2016 for those aged 15–39 years; 71·2% of the suicide deaths among women and 57·7% among men were in this age group. If the trends observed up to 2016 continue, the probability of India achieving the SDG SDR reduction target in 2030 is zero, and the majority of the states with 81·3% of India's population have less than 10% probability, three states have a probability of 10·3–15·0%, and six have a probability of 25·1–36·7%.

**Interpretation:**

India's proportional contribution to global suicide deaths is high and increasing. SDR in India is higher than expected for its Socio-Demographic Index level, especially for women, with substantial variations in the magnitude and men-to-women ratio between the states. India must develop a suicide prevention strategy that takes into account these variations in order to address this major public health problem.

**Funding:**

Bill & Melinda Gates Foundation; and Indian Council of Medical Research, Department of Health Research, Ministry of Health and Family Welfare, Government of India.

## Introduction

An estimated 817 000 suicide deaths occurred globally in 2016, accounting for 1·5% of all deaths, with a global suicide death rate (SDR) of 11 per 100 000 population (seven per 100 000 for women and 15 per 100 000 for men).^1^, ^2^ Young and middle-aged adults die of suicides predominantly; and suicide is the second leading cause of death worldwide among those aged 15–29 years, and the third leading cause among those aged 15–39 years.^1^, ^3^, ^4^ With increasing realisation of the public health importance of suicides, the UN Sustainable Development Goals (SDGs) include a one-third reduction in SDR from 2015 to 2030.^5^

India accounts for a large proportion of all suicide deaths globally.^1^ With 18% of the world's population living in India and 42% of the population aged 15–39 years,^6^ addressing suicides in India is imperative to making a global difference in the burden of suicides. A description of the epidemiology of suicide deaths in the states of India was reported from a nationally representative survey of causes of death during 2001–03, using verbal autopsy.^7^ Some studies^8^, ^9^ have described suicide trends for the states of India as reported by the National Crimes Record Bureau of India, which is known to have under-reporting of suicides. A comprehensive analysis and understanding of trends in SDRs over time for India and its states is however not readily available. The states of India have substantial cultural, social, and economic variations, and many states have populations as large as mid-size or large countries. It is important, therefore, to understand the time trends of suicide in each state to inform effective suicide prevention policies and programmes. As highlighted in the findings from the India State-Level Disease Burden Initiative, the states in India are at varying levels of epidemiological transition, which has resulted in wide heterogeneity in disease burden across the states.^10^ In this Article, we aimed to report time trends of suicide deaths, and the heterogeneity in its distribution between the states of India from 1990 to 2016.

**Research in context**

**Evidence before this study**

Existing evidence suggests that India accounted for more than a quarter of all suicide deaths globally in 2016. A previous study reported suicide death rates for India and its states using verbal autopsy data from 2001 to 2003, and some studies assessed trends of suicide rates in the states as reported by the National Crimes Record Bureau of India that is known to have under-reporting. We searched PubMed and publicly available reports for estimates of suicide deaths across the states of India using the search terms “death”, “cause of death”, “epidemiology”, “India”, “mortality”, “self-harm”, “suicide”, and “trends” on March 31, 2018, without language or publication date restrictions. We found a variety of data related to suicide in India and several states, but no studies that comprehensively described population-based estimates of suicide death trends in every state of India over a long period.

**Added value of this study**

This study provides a comprehensive assessment of the burden of suicide deaths from 1990 to 2016 for each state of India using methods that are comparable over time and between states, with a focus on sex and state differentials in suicide death rates. The findings highlight that suicide death rates were higher in India than the global average in 2016, and most states had rates much higher than would be expected for their Socio-demographic Index level. These findings suggest that if the trends observed up to 2016 continue India and most of its states are unlikely to achieve the Sustainable Development Goal target of one-third reduction in suicide death rate from 2015 to 2030.

**Implications of all the available evidence**

This analysis provides a comprehensive assessment of the trends of suicide deaths in every state of India over the past quarter century. These time trend data provide a reference that can be used to develop suicide prevention plans suitable for each state within a national suicide prevention strategy, which is needed to reduce suicide deaths across India.

## Methods

### Overview

The India State-Level Disease Burden Initiative has recently reported the overall trends of diseases, injuries, and risk factors from 1990 to 2016 for every state of India.^10^ This analysis was done as part of the Global Burden of Diseases, Injuries, and Risk Factors Study (GBD) 2016, which estimated disease burden of 333 diseases and injuries and 84 risk factors using all accessible data from multiple sources. The India State-Level Disease Burden Initiative was supported by the efforts of several expert groups and a vast network of collaborators to identify and access a variety of data sources, assess their scope and quality for inclusion, and participate in the analysis and interpretation of the findings. The Health Ministry Screening Committee at the Indian Council of Medical Research and the ethics committee of the Public Health Foundation of India approved the work of this initiative. A detailed description of metrics and analytical approaches used in GBD 2016 has been reported elsewhere.^2^, ^10^, ^11^, ^12^

### Estimation of cause-specific mortality

The methods used to estimate causes of mortality for India in GBD 2016 have been described elsewhere.^10^ The methods relevant to this paper are summarised here and described in the appendix (pp 3–5). The major data sources for cause-specific mortality estimation in India include sample registration and vital registration, medically certified causes of death, and verbal autopsy studies (appendix pp 6–11). Verbal autopsy cause of death data were included for 455 460 deaths covering every state of India from 2004 to 2013, as part of the Sample Registration System of India.^10^ The quality and comparability of the cause of death data were assessed and enhanced through multiple steps, which have been reported previously.^2^, ^10^, ^11^, ^13^ In the raw cause of death data used for estimating suicide deaths, the deaths assigned to ill-defined or improbable causes of death were redistributed to the probable true underlying causes of death using various methods described in detail previously.^2^ Furthermore, data that were reported in aggregated categories were split into estimates of age-specific and sex-specific deaths using the observed global pattern of deaths for a cause by age and sex and the local age-sex distribution of the population.

The Cause of Death Ensemble model was used to evaluate a large number of potential models that apply different functional forms, such as mixed effects models and space-time Gaussian process regression models, to death rates or cause fractions with varying combinations of predictive covariates. An ensemble of models that performs best on out-of-sample predictive validity tests were selected for each cause of death. Finally, the CoDCorrect algorithm was used to rescale deaths for each cause to ensure that deaths from all individual causes sum to the number of deaths from all causes generated from the demographic analysis. The International Classification of Diseases (ICD) was used to classify injuries including suicides (ICD-10 codes X60–X64.9, X66–X84.9, and Y87.0).^13^, ^14^ Because cause of death data in India before 2000 were generally scarce, the estimates before this period were based largely on covariates (appendix p 12).^2^, ^10^ GBD uses covariates, which are variables that have an established association with the outcome of interest, to arrive at the best possible estimates when data for the outcome are scarce but the data for covariates are available.

### Analysis presented in this paper

We present trends in SDR from 1990 to 2016 for both sexes for each state of India to highlight the heterogeneity across the country. Findings are presented for 31 geographical units in India: 29 states, union territory of Delhi, and union territories other than Delhi (combining the six smaller union territories of Andaman and Nicobar Islands, Chandigarh, Dadra and Nagar Haveli, Daman and Diu, Lakshadweep, and Puducherry). The states of Chhattisgarh, Uttarakhand, and Jharkhand were established from existing larger states in 2000, and the state of Telangana was established in 2014. For trends from 1990 onwards, the data for these four new states were disaggregated from their parent states on the basis of data from the districts that now constitute these states. The findings are presented in four groups of states on the basis of their epidemiological transition level (ETL) as described previously.^10^ Briefly, ETL state groups were defined on the basis of the ratio of DALYs from communicable, maternal, neonatal and nutritional diseases to those from non-communicable diseases and injuries combined in 2016, with a relatively lower ratio indicating higher ETL: low ETL state group (ratio 0·56–0·75), lower-middle ETL (0·41–0·55), higher-middle ETL (0·31–0·40), and high ETL (less than 0·31). We have previously reported that epidemiological transition ratios of the states of India have a significant inverse relationship with the Socio-demographic Index (SDI) computed by GBD based on income, education and fertility levels, which indicates broad correspondence of the ETL groups with sociodemographic development levels.^10^

We estimated crude and age-standardised SDR for women and men for each state of India from 1990 to 2016. Crude estimates provide the actual situation in each state that is useful for policy makers, and age-standardised estimates allow comparisons over time and between states after adjusting for the differences in the age structure of the population. Age-standardised SDRs were based on the GBD global reference population.^10^ We assessed the ratio of the observed SDR among women and men in each state of India to that expected globally in geographies at a similar level of Socio-Demographic Index.^12^ We examined age-specific SDRs and suicide deaths among men and women and their change from 1990 to 2016. We assessed India's contribution to the global suicide deaths as reported in GBD 2016.^1^ We analysed the trends of the men-to-women ratio of crude SDR for each state, the ETL state groups, and India from 1990 to 2016.

We projected the SDR for India and for each state up to 2030 based on the trends from 1990 to 2016, in order to ascertain the probability of meeting the SDG target of a one-third reduction in age-standardised SDR from 2015 to 2030 for both sexes combined.^15^ The annual rate of change used in the projection of age-standardised SDR for each state was calculated using a weight function that gave higher weight to the more recent trends in each state. The detailed methods used for these projections, including the out-of-sample predictive validity test, are described in the appendix (p 5) and elsewhere.^15^ Estimates are reported with 95% uncertainty intervals (UIs) where relevant, which were based on 1000 draws for each estimate (appendix p 5).

### Role of the funding source

Some staff of the Indian Council of Medical Research are coauthors on this paper as they contributed to various aspects of the study and this analysis. The other funder of the study had no role in the study design, data collection, data analysis, data interpretation, or writing of this paper. The corresponding author had full access to all of the data in the study and had final responsibility for the decision to submit for publication.

## Results

**Figure 1:**
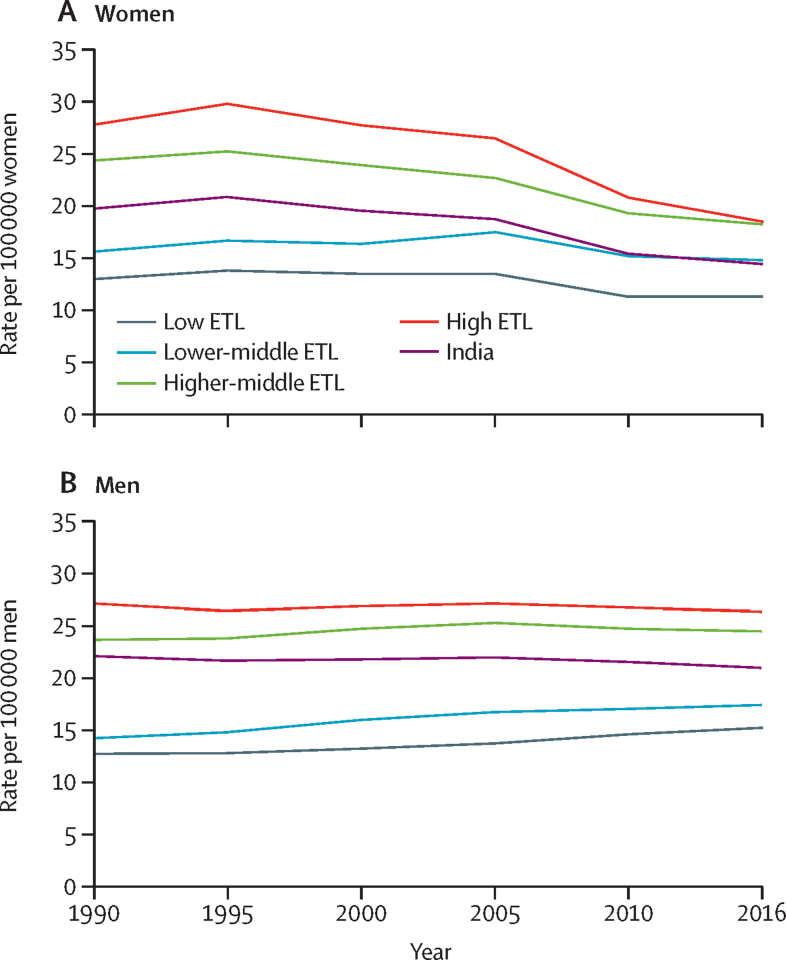
Trends of age-standardised suicide death rates among women and men in the ETL state groups and India, 1990–2016 ETL=epidemiological transition level.

Suicide deaths in India increased from 164 404 (95% UI 134 118–180 940) in 1990 to 230 314 (194 058–250 260) in 2016, an increase of 40·1% (95% UI 25·8–65·1). India had 864 million (16·4%) of the global population in 1990, and 81 040 (25·3%) of the 320 567 global suicide deaths among women and 83 365 (18·7%) of the 445 476 global suicide deaths among men.^1^ In 2016, India had 1316 million (17·8%) of the global population, but its contribution to suicides increased to 94 380 (36·6%) of the 257 624 global suicide deaths among women and 135 934 (24·3%) of the 559 523 global suicide deaths among men.^1^ Suicide was the ninth leading cause of death in India in 2016 with an age-standardised SDR of 17·9 (95% UI 15·0–19·4) per 100 000 population, accounting for 2·35% of all deaths with 94 380 (95% UI 84 002–104 274) deaths in women and 135 934 (94 305–151 239) in men. Age-standardised SDR among women reduced from 1990 to 2016 by 26·7% (95% UI 7·6–40·3), but did not change significantly during this period among men (−4·9%, 95% UI −25·2 to 50·0; Table 1, Table 2; figure 1).

**Table 1:** Change in crude and age-standardised SDRs among women in the states of India grouped by ETL, 1990–2016

		**SDR per 100 000 women in 1990 (95% UI)**		**SDR per 100 000 women in 2016 (95% UI)**		**Percentage change, 1990–2016 (95% UI)**	
		Crude	Age standardised	Crude	Age standardised	Crude	Age standardised
India (1316 million)		19·4 (16·1–22·9)	20·0 (16·5–23·5)	14·9 (13·2–16·4)	14·7 (13·1–16·2)	−23·5 (−38·2 to −3·2)	−26·7 (−40·3 to −7·6)
Low ETL (626 million)		13·3 (10·8–16·1)	14·0 (11·6–16·9)	11·4 (9·7–13·2)	11·6 (9·9–13·5)	−14·3 (−33·9 to 11·6)	−17·2 (−35·2 to 6·7)
	Bihar	5·5 (4·2–7·1)	6·1 (4·8–7·9)	6·2 (4·9–7·9)	7·2 (5·6–9·1)	13·5 (−18·0 to 59·0)	17·1 (−13·3 to 61·6)
	Jharkhand	9·8 (7·0–13·5)	10·9 (8·0–14·8)	8·0 (5·6–10·7)	8·5 (5·9–11·2)	−18·4 (−48·8 to 25·2)	−22·4 (−49·9 to 15·6)
	Uttar Pradesh	16·1 (11·6–20·9)	16·9 (12·4–21·8)	14·0 (11·1–17·8)	14·1 (11·2–17·7)	−12·6 (−39·3 to 30·6)	−16·6 (−40·8 to 22·7)
	Rajasthan	7·6 (5·6–9·9)	8·3 (6·1–10·7)	8·3 (6·7–10·4)	8·4 (6·8–10·5)	10·4 (−22·4 to 63·5)	1·6 (−26·7 to 47·2)
	Meghalaya	3·5 (2·4–4·9)	3·7 (2·5–5·0)	3·5 (2·7–4·4)	3·4 (2·7–4·3)	−1·6 (−37·7 to 69·7)	−7·7 (−39·3 to 53·5)
	Assam	20·0 (14·3–27·2)	20·5 (15·0–27·6)	13·8 (10·5–18·2)	13·3 (10·1–17·3)	−30·9 (−55·6 to 11·0)	−35·0 (−57·5 to 1·6)
	Chhattisgarh	12·5 (9·3–16·4)	14·3 (10·8–18·7)	11·8 (9–15·3)	11·6 (8·9–14·9)	−5·6 (−37·3 to 47·0)	−18·9 (−45·1 to 23·6)
	Madhya Pradesh	16·6 (12·0–21·5)	17·0 (12·6–21·7)	13·4 (10·3–17)	13·5 (10·5–16·9)	−19·3 (−45·0 to 19·3)	−20·8 (−45·1 to 15·6)
	Odisha	18·4 (12·9–24·4)	18·4 (13·0–24·2)	13·2 (10·1–17)	12·9 (9·9–16·4)	−28·3 (−52·6 to 11·6)	−30·0 (−52·8 to 7·3)
Lower-middle ETL (92 million)		15·8 (11·9–19·5)	15·8 (12·0–19·6)	14·9 (12·6–17·8)	14·3 (12·2–17)	−5·4 (−29·0 to 30·8)	−9·4 (−31·6 to 24·1)
	Arunachal Pradesh	16·9 (12·3–22·5)	18·8 (13·8–25·1)	13·2 (10–17·5)	13·8 (10·5–18)	−21·9 (−48·1 to 26·0)	−26·6 (−50·7 to 14)
	Mizoram	3·6 (2·4–4·8)	3·6 (2·5–4·8)	2·6 (1·9–3·3)	2·5 (1·8–3·2)	−28·3 (−53·7 to 14·9)	−32·7 (−55·0 to 4·5)
	Nagaland	4·4 (2·7–6·4)	4·3 (2·7–6·1)	2·7 (1·9–3·6)	2·6 (1·9–3·4)	−38·4 (−63·7 to 13·5)	−40·0 (−63·7 to 5·5)
	Uttarakhand	17·9 (12·9–23·4)	20·5 (14·9–26·6)	11·8 (9–15·3)	11·3 (8·7–14·5)	−34·0 (−56·7 to 5·4)	−44·8 (−63·4 to −12·1)
	Gujarat	15·4 (11·1–19·7)	15·1 (11·0–19·3)	16·0 (13·3–19·4)	15·4 (12·8–18·7)	4·1 (−24·6 to 51·4)	2·4 (−25·1 to 48·2)
	Tripura	28·3 (21·3–36·2)	28·2 (21·3–36·4)	21·4 (16·7–27)	20·3 (15·7–25·4)	−24·4 (−46·3 to 9·5)	−28·0 (−48·6 to 1·7)
	Sikkim	14·3 (9·1–23·3)	14·2 (9·2–23·0)	8·2 (6·3–11·1)	8·1 (6·2–10·7)	−42·5 (−69·2 to 7·0)	−43·0 (−69·2 to 3·3)
	Manipur	9·4 (6·5–12·8)	9·6 (6·7–12·9)	8·9 (6·9–11·6)	8·4 (6·5–10·8)	−5·1 (−38·3 to 51·3)	−12·2 (−41·1 to 36·7)
Higher-middle ETL (446 million)		24·5 (19·6–29·4)	24·9 (19·9–29·7)	18·4 (16·3–20·5)	17·3 (15·4–19·3)	−25·0 (−40·8 to −0·9)	−30·2 (−44·6 to −8·1)
	Haryana	14·8 (10·9–19·0)	15·6 (11·7–19·8)	10·5 (8·4–13·2)	10·0 (8–12·4)	−28·8 (−50·1 to 1·9)	−35·6 (−54·1 to −7·8)
	Delhi	8·1 (5·6–11·0)	8·0 (5·6–10·6)	5·7 (4·2–7·4)	5·2 (3·9–6·6)	−30·5 (−55·0 to 14·0)	−36·0 (−57·3 to 2·2)
	Telangana	29·0 (19·8–39·0)	29·5 (20·2–39·5)	19·8 (15·3–25)	18·8 (14·5–23·7)	−31·6 (−55·0 to 9·4)	−36·3 (−57·8 to 2·0)
	Andhra Pradesh	27·9 (20·2–36·7)	27·8 (20·2–36·4)	21·0 (16·9–25·8)	19·8 (15·9–24·5)	−24·9 (−48·0 to 11·7)	−28·9 (−50·3 to 6·3)
	Jammu and Kashmir	13·1 (9·4–17·8)	13·5 (9·7–18·1)	8·9 (6·8–11·8)	8·4 (6·5–11)	−32·2 (−55·9 to 7·9)	−37·8 (−59·1 to −2·1)
	Karnataka	26·6 (19·3–33·8)	26·7 (19·1–33·8)	25·1 (20·1–31·1)	23·5 (19–28·9)	−5·9 (−32·7 to 37·0)	−11·8 (−36·0 to 28·1)
	West Bengal	32·5 (24·3–40·9)	33·2 (25·1–41·7)	22·1 (17·8–27)	20·6 (16·5–25·2)	−31·9 (−51·2 to −1·7)	−37·8 (−55·0 to −10·9)
	Maharashtra	18·0 (12·7–23·7)	18·5 (13·2–24·1)	14·8 (12–17·9)	14·0 (11·4–16·9)	−18·1 (−43·1 to 26·2)	−24·1 (−46·8 to 16·3)
	Union territories other than Delhi	22·2 (15·9–28·5)	21·5 (15·3–27·9)	15·1 (11·5–19·4)	14·1 (10·9–17·9)	−31·8 (−54·1 to 6·8)	−34·5 (−55·9 to 1·1)
High ETL (152 million)		28·1 (22·3–33·6)	27·5 (22·0–33·1)	18·8 (16·2–21·9)	17·7 (15·2–20·6)	−33·1 (−48·4 to −9·5)	−35·7 (−50·4 to −13·1)
	Himachal Pradesh	14·0 (8·5–19·5)	14·0 (8·6–19·5)	8·9 (7·1–11·3)	8·4 (6·7–10·5)	−36·0 (−60·0 to 15·5)	−40·3 (−62·6 to 6·5)
	Punjab	9·5 (7·0–12·4)	9·4 (7·1–12·1)	7·7 (6–9·7)	7·0 (5·5–8·7)	−18·6 (−43·8 to 18·0)	−25·4 (−48·3 to 7·4)
	Tamil Nadu	41·9 (32·3–52·0)	41·4 (32·2–51·3)	26·9 (22·1–32·6)	25·3 (20·9–30·6)	−35·8 (−52·9 to −7·7)	−38·7 (−55·0 to −12·1)
	Goa	16·8 (11·4–22·2)	15·8 (10·6–0·7)	10·7 (9·3–13)	10·5 (9·1–12·7)	−36·1 (−54·7 to 1·5)	−33·4 (−52·5 to 6·2)
	Kerala	16·8 (12·6–20·8)	16·1 (12·2–19·9)	13·2 (11·4–15·4)	12·6 (10·9–14·6)	−21·3 (−37·7 to 3·3)	−21·6 (−38·0 to 3·7)

Population in 2016 given in parenthesis. ETL=epidemiological transition level. SDR=suicide death rate. UI=uncertainty interval.

**Table 2:** Change in crude and age-standardised SDRs among men in the states of India grouped by ETL, 1990–2016

		**SDR per 100 000 men in 1990 (95% UI)**		**SDR per 100 000 men in 2016 (95% UI)**		**Percentage change, 1990–2016 (95% UI)**	
		Crude	Age standardised	Crude	Age standardised	Crude	Age standardised
India (1316 million)		18·6 (12·1–22·8)	22·3 (14·4–27·4)	19·9 (13·8–22·2)	21·2 (14·6–23·6)	6·9 (−15·8 to 28·9)	−4·9 (−25·2 to 15·0)
Low ETL (626 million)		12·7 (10·3–16·0)	15·5 (12·5–19·6)	15·4 (13·4–17·9)	17·3 (15·1–20·1)	21·1 (−5·4 to 50·4)	12·1 (−12·1 to 39·6)
	Bihar	5·5 (3·5–12·7)	7·1 (4·5–16·4)	8·5 (6·5–12·8)	10·5 (8·1–15·2)	54·5 (−8·5 to 141·8)	49·2 (−15·4 to 133·5)
	Jharkhand	9·0 (6·3–15·5)	11·8 (8·3–20·7)	9·8 (7·2–16·7)	11·5 (8·5–19·7)	8·8 (−26·2 to 58·7)	−2·7 (−33·3 to 41·1)
	Uttar Pradesh	13·6 (10·3–17·9)	16·4 (12·3–21·7)	15·4 (12·1–18·8)	17·3 (13·7–21·2)	13·1 (−20·1 to 59·5)	5·9 (−25·0 to 50·2)
	Rajasthan	11·3 (8·6–16·0)	14·0 (10·7–19·6)	15·3 (12·1–19·6)	17·2 (13·7–22·3)	34·4 (−5·7 to 87·9)	22·7 (−14·0 to 69·6)
	Meghalaya	9·5 (7·0–13·5)	12·0 (8·9–17·1)	12·5 (9·8–18·1)	14·6 (11·6–21·4)	31·1 (−9·0 to 84·9)	21·3 (−14·4 to 70·8)
	Assam	17·8 (13·7–22·8)	20·7 (16·1–26·8)	18·7 (14·4–24·7)	19·6 (15·2–26)	5·0 (−27·2 to 45·3)	−5·3 (−33·4 to 29·6)
	Chhattisgarh	19·8 (10·8–25·9)	26·6 (14·3–35·0)	27·7 (17·2–35·3)	29·8 (18·3–37·9)	40·1 (0·4 to 97·3)	11·9 (−19·2 to 57·4)
	Madhya Pradesh	16·2 (11·5–20·7)	19·1 (13·8–24·3)	21·3 (15·6–26·4)	23·2 (17–28·6)	32·0 (−3·9 to 78·9)	21·5 (−11·1 to 64·5)
	Odisha	15·5 (11·9–21·2)	17·7 (13·5–24·3)	16·7 (12·9–23·3)	17·1 (13·2–24)	7·6 (−25·3 to 50·3)	−3·3 (−32·6 to 34·8)
Lower-middle ETL (92 million)		14·3 (11·4–17·7)	17·0 (13·8–21·0)	17·5 (14·1–20·6)	18·1 (14·7–21·2)	22·4 (−9·6 to 57·4)	6·4 (−20·8 to 36·1)
	Arunachal Pradesh	15·1 (9·9–19·3)	19·0 (12·1–24·4)	17·7 (12·8–22·5)	21·1 (14·7–26·7)	17·1 (−16·5 to 62·9)	10·8 (−21·4 to 54·3)
	Mizoram	8·7 (6·3–14·4)	10·5 (7·5–17·9)	10·5 (7·9–17·2)	11·4 (8·6–19·1)	20·5 (−17·9 to 67·3)	8·6 (−24·3 to 50·0)
	Nagaland	5·4 (3·4–14·6)	6·5 (4·0–17·5)	6·6 (4·4–17·1)	7·4 (4·9–19·2)	21·5 (−16·9 to 74·0)	13·5 (−21·2 to 63·0)
	Uttarakhand	13·3 (9·3–18·4)	18·2 (12·8–25·0)	13·6 (10·3–18·9)	14·6 (11·2–20·5)	2·1 (−35·1 to 59·3)	−20·0 (−49·1 to 24·2)
	Gujarat	13·9 (11·0–17·7)	16·1 (12·8–20·4)	17·2 (13·7–20·8)	17·6 (14·1–21·6)	24·1 (−10·8 to 64·9)	9·3 (−20·9 to 43·9)
	Tripura	29·3 (12·2–40·0)	33·0 (13·7–45·4)	38·7 (15·6–50·8)	38·6 (15·5–50·4)	32·1 (−2·6 to 81·1)	16·8 (−14·1 to 61·1)
	Sikkim	12·7 (9·0–17·2)	15·1 (10·8–20·7)	15·3 (11·6–21·4)	16·5 (12·6–23)	20·3 (−15·4 to 87·1)	9·2 (−23·3 to 66·9)
	Manipur	14·4 (10·1–19·3)	17·8 (12·5–24·3)	16·9 (12–21·6)	18·1 (12·9–23)	18·0 (−18·8 to 70·5)	1·4 (−30·0 to 48·6)
Higher-middle ETL (446 million)		23·8 (12·4–30·2)	27·6 (14·4–35·2)	24·7 (14·2–28·6)	24·7 (14·1–28·6)	3·8 (−20·5 to 30·8)	−10·5 (−31·8 to 12·6)
	Haryana	16·2 (11·7–21·0)	19·8 (14·3–25·6)	19·9 (15·4–24·1)	20·7 (16·1–25)	23·0 (−13·0 to 78·1)	4·6 (−26·3 to 53·9)
	Delhi	9·1 (6·7–15·2)	10·3 (7·5–17·5)	9·1 (6·6–14·5)	9·2 (6·7–14·8)	0·1 (−30·0 to 41·1)	−10·8 (−37·9 to 24·3)
	Telangana	24·4 (11·1–33·9)	29·3 (13·3–40·6)	24·8 (12·3–32·9)	25·2 (12·5–33·3)	1·7 (−32·8 to 52·8)	−13·9 (−42·3 to 29·5)
	Andhra Pradesh	25·7 (11·3–35·2)	30·3 (13·0–41·2)	28·8 (14·2–36·2)	29·1 (14·3–36·4)	12·3 (−20·3 to 54·3)	−3·9 (−31·5 to 31·0)
	Jammu and Kashmir	9·0 (6·3–16·7)	10·9 (7·6–20·2)	8·6 (6·4–16·5)	9·1 (6·8–17·4)	−4·0 (−35·8 to 35·0)	−16·2 (−44·2 to 16·1)
	Karnataka	32·7 (13·1–44·3)	38·3 (15·0–52·2)	36·1 (16–46·4)	36·0 (15·6–46·7)	10·4 (−23·8 to 53·1)	−5·9 (−34·9 to 30·0)
	West Bengal	26·7 (11·6–35·9)	30·4 (13·4–40·8)	25·0 (12·4–31·8)	24·5 (12·2–30·9)	−6·4 (−32·0 to 29·0)	−19·3 (−40·8 to 11·4)
	Maharashtra	20·1 (12·6–26·8)	22·9 (14·5–30·2)	22·4 (15–27·3)	22·2 (14·8–27·1)	11·2 (−21·9 to 53·8)	−3·1 (−32·4 to 32·7)
	Union territories other than Delhi	19·4 (10·7–25·3)	22·3 (11·9–29·2)	19·3 (12–24·6)	18·9 (11·6–23·9)	−0·6 (−29·5 to 44·0)	−15·4 (−39·9 to 24·1)
High ETL (152 million)		27·3 (13·1–36·3)	31·9 (14·8–42·6)	26·5 (13·5–32·1)	25·8 (13·1–31·3)	−3·1 (−27·3 to 25·1)	−19·1 (−39·5 to 5·5)
	Himachal Pradesh	12·1 (8·8–16·7)	14·3 (10·3–19·8)	16·4 (12·8–20·9)	16·1 (12·6–20·4)	35·6 (−10·9 to 100·3)	12·2 (−25·8 to 66·1)
	Punjab	9·7 (6·6–18·2)	11·5 (7·9–21·6)	11·1 (8·3–19)	11·0 (8·3–18·8)	14·6 (−20·8 to 71·7)	−4·5 (−34·0 to 41·4)
	Tamil Nadu	34·4 (14·5–48·0)	39·6 (16·3–55·2)	32·8 (14·7–42·1)	31·7 (14·2–40·6)	−4·9 (−32·8 to 30·9)	−19·8 (−43·4 to 10·6)
	Goa	13·0 (9·9–16·9)	14·5 (11·1–18·9)	12·0 (9·6–15·2)	11·5 (9·3–14·5)	−7·8 (−35·7 to 28·4)	−20·9 (−44·9 to 9·2)
	Kerala	29·7 (10·8–40·1)	35·2 (12·0–48·0)	29·5 (10·1–37·9)	28·3 (9·9–35·8)	−0·8 (−28·2 to 33·8)	−19·7 (−41·4 to 8·6)

Population in 2016 given in parentheses. ETL=epidemiological transition level. SDR=suicide death rate. UI=uncertainty interval.

Suicide deaths in 2016 accounted for 3·07% (95% UI 2·21–3·44) of all deaths in the high ETL state group, 3·02% (2·33–3·32) in the higher-middle ETL state group, 2·35% (2·13–2·55) in the lower-middle ETL state group, and 1·74% (1·58–1·94) in the low ETL state group. The high and higher-middle ETL state groups had significant declines in age-standardised SDR for women from 1990 to 2016, but no significant change for men (Table 1, Table 2; figure 1). The low and lower-middle ETL state groups did not have a significant change in SDR during this period among women or men. In 2016, the high and higher-middle ETL state groups continued to have higher SDRs than the low and lower-middle ETL state groups for both women and men. However, there were variations between the states within the ETL state groups. Significant declines in age-standardised SDR were observed for women from 1990 to 2016 only in Tamil Nadu in the high ETL group, in Haryana, Jammu and Kashmir, and West Bengal in the higher-middle ETL group, and in Uttarakhand in the lower-middle ETL group (table 1). No state had a significant change in age-standardised SDR for men during this period (table 2).

**Figure 2:**
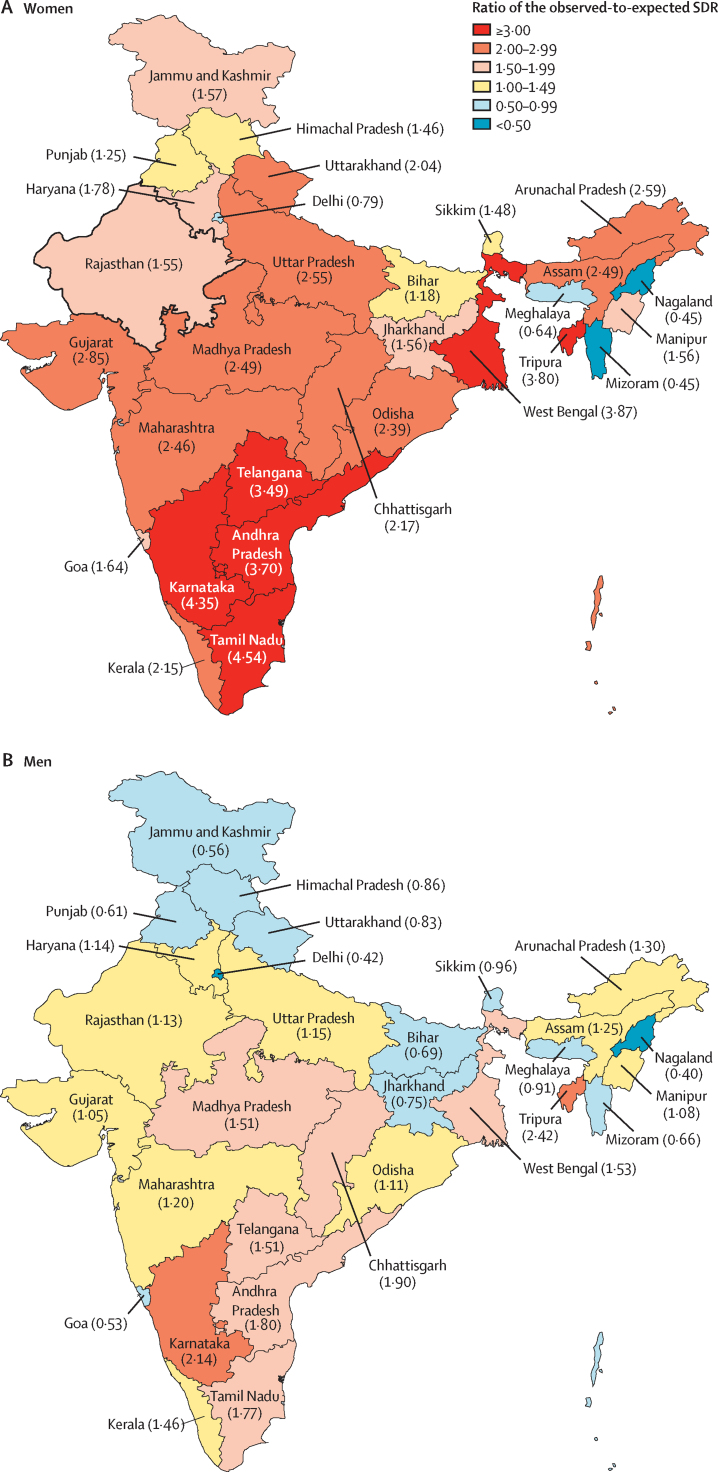
Ratio of the observed SDR among women and men in the states of India to that expected for their Socio-Demographic Index level, 2016 Data in parentheses are observed-to-expected ratio of SDRs. SDR=suicide death rate.

The age-standardised SDR of 14·7 (95% UI 13·1–16·2) per 100 000 women in India in 2016 was 2·1 times higher in India than the global average in 2016, and the observed-to-expected ratio was 2·74. The highest SDR among women in 2016 was in the states of Tamil Nadu and Karnataka, followed by West Bengal, Tripura, Andhra Pradesh, and Telangana (table 1). These six states had age-standardised SDR of more than 18 per 100 000 women; only three countries in the world had SDRs higher than this level among women in 2016 (appendix p 13). The SDR in women ranged ten-fold between the states of India. Even within the group of eight states in the northeast region of India, this ranged eight-fold. The observed-to-expected ratio of SDR among women ranged from 0·45 to 4·54 in 2016 (figure 2). Six states with 25·7% of India's population had an observed-to-expected ratio of more than three, ten states with 48·8% of India's population had an observed-to-expected ratio of between two and three, and six states with 11·8% of India's population had an observed-to-expected ratio of between 1·5 and two. The numbers of suicide deaths among women in each state are shown in the appendix (p 14).

The age-standardised SDR of 21·2 (95% UI 14·6–23·6) per 100 000 men in India in 2016 was 1·4 times higher in India than the global average in 2016, and the observed-to-expected ratio was 1·31. The eight states with the highest SDR among men in 2016 (above 24 per 100 000 men) included the six states that had the highest SDR among women (ie, Tamil Nadu, Karnataka, West Bengal, Tripura, Andhra Pradesh, and Telangana) as well as Kerala and Chhattisgarh (table 2). The crude SDR in men ranged six-fold between the states of India. The highest and the lowest crude SDR for men were within the group of eight states in the northeast region of India. The observed-to-expected ratio of SDR in men in 2016 among the states of India ranged from 0·40 in Nagaland to 2·42 in Tripura (figure 2). Eight states with 33·9% of India's population had an observed-to-expected ratio of between 1·5 and three, ten states with 48·0% of India's population had an observed-to-expected ratio between one and 1·5, and 12 states with 17·7% of India's population had an observed-to-expected ratio of less than one. The numbers of suicide deaths among men in each state are shown in the appendix (p 14).

**Figure 3:**
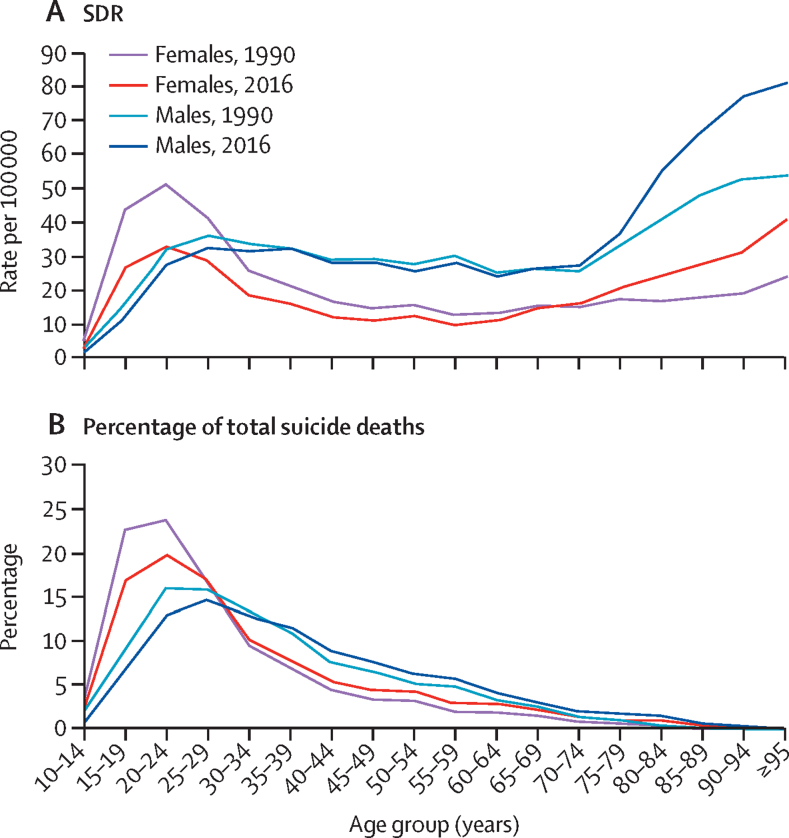
Age-specific SDR and the percentage of total suicide deaths in each age group among females and males in India, 1990 and 2016 SDR=suicide death rate. ETL=epidemiological transition level.

The age-specific SDR among girls and women decreased significantly from 1990 to 2016 for those aged 10–34 years, but increased significantly for those older than 80 years (figure 3; appendix p 15). Among men, the only significant change during this period was an increase in the SDR for those older than 80 years. In 2016, the highest SDR among younger women were in the age groups of 15–29 years (range 26·7–33·1 per 100 000 women), and SDR among older women increased from 20·9 per 100 000 women in the age group of 75–79 years to 40·6 per 100 000 women in those 95 years or older. Among men, the SDR in 2016 was in a similar range between ages 20–74 years (24·5–33·1 per 100 000 men), and then increased in the older age groups to as high as 80·8 per 100 000 men in those 95 years or older. The age distribution of SDR was generally similar in the ETL groups in 2016 for both men and women (appendix p 21). A large proportion of the suicide deaths in India in 2016 was in the age groups 15–39 years in both women (71·2%) and men (57·7%), although these proportions were somewhat lower than those in 1990 among both women (79·8%) and men (65·4%; figure 3; appendix p 16). Suicide was the leading cause of death for those in the age groups of 15–29 years and 15–39 years in India in 2016 for both sexes combined (table 3). Suicide was the leading contributor to deaths in these age groups among women, and the second leading contributor among men in India. Suicide deaths ranked first among all causes of death in women aged 15–29 years in 26 of the 31 states, and in women aged 15–39 years in 24 states; for men, suicide was the leading cause of death in nine states for those aged 15–29 years and ten states in those aged 15–39 years (appendix p 17).

**Table 3:** Percentage of total deaths due to suicide in young adults by sex in India, 2016

	**Percentage of total deaths due to suicide (95% UI)**	Rank of suicide deaths*
**Both sexes combined**		
15–29 years	16·9 (15·1–18·5)	1
15–39 years	13·0 (11·4–14·2)	1
**Females**		
15–29 years	20·3 (18·0–22·5)	1
15–39 years	15·3 (13·6–16·8)	1
**Males**		
15–29 years	14·3 (10·7–16·0)	2
15–39 years	11·6 (8·4–12·8)	2

UI=uncertainty interval.

* Rank of suicide deaths among all individual causes of death.

The men-to-women ratio of crude SDR increased from 0·96 in 1990 to 1·34 in 2016, which was due to the decrease in SDR among women during this period especially in the younger age groups. Even with this decrease, the men-to-women ratio of crude SDR continued to be less than one up to 24 years of age in 2016, but was more than one in the older age groups (appendix p 18). In 1990, the men-to-women ratio of crude SDR was similar in all ETL state groups, but this ratio was smaller in the lower-middle ETL state group than the other ETL state groups in 2016 because of its relatively lower increase in this state group during this period (appendix p 18). The men-to-women ratio of SDR in India was lower than the global average in both 1990 and 2016, which also increased from 1·37 to 2·14 during this period.

The men-to-women ratio of SDR increased in every state of India from 1990 to 2016, although most of this increase was during the 2005 to 2016 period (appendix p 19). There were however wide variations in this ratio between the states, ranging from 0·97 to 4·11. As compared with the 1·34 men-to-women ratio of SDR for India in 2016, Jammu and Kashmir, Gujarat, Uttar Pradesh, Goa, West Bengal and Uttarakhand had ratios of 1·15 or less; whereas Mizoram, Meghalaya, Nagaland, Chhattisgarh, Kerala, Manipur, Haryana, Sikkim, Himachal Pradesh, Rajasthan, and Tripura had ratios of 1·80 or more, which included six of the eight states in northeast India.

**Figure 4:**
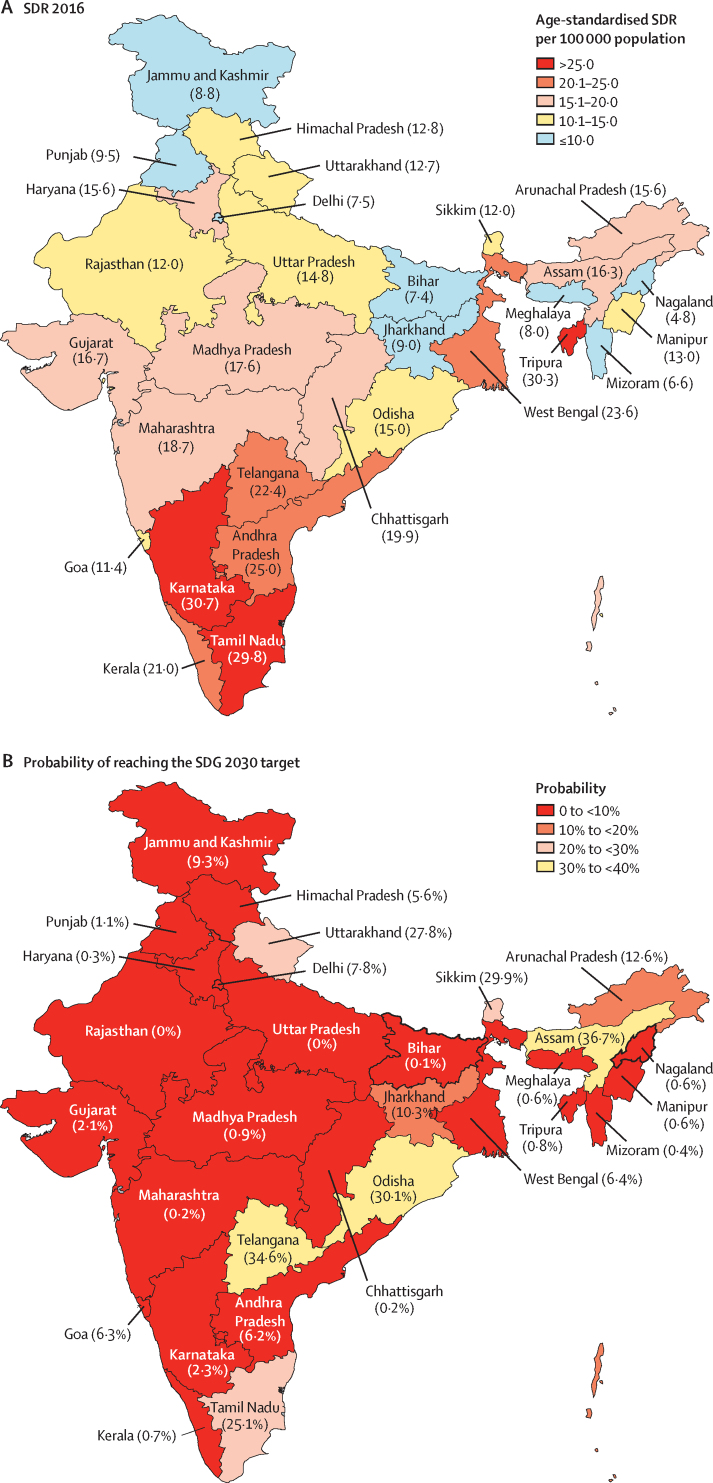
SDR for both sexes combined in 2016 and the probability of reaching the SDG 2030 target in the states of India Data in parentheses are SDR or probability of reaching the SDG 2030 target. SDR=suicide death rate. SDG=Sustainable Development Goal.

If the trends of SDR observed up to 2016 continue, India is projected to have an age-standardised SDR of 15·7 (95% UI 13·0–15·1) per 100 000 population for both sexes combined in 2030. This projected estimate falls short of the SDG target of 12·1 per 100 000 population, based on the one-third reduction of SDR from 2015 to 2030, giving India a zero probability of meeting this target (appendix p 20). The vast majority of states in India that have 81·3% of the country's population have less than 10% probability of meeting the SDG 2030 SDR reduction target, three states have a probability of 10·3–15·0%, and six have a probability of 25·1–36·7% (figure 4; appendix p 20).

## Discussion

India had 17·8% of the global population in 2016, but accounted for 36·6% of the global suicide deaths among women and 24·3% among men. The proportion of global suicide deaths in India has increased since 1990 for both sexes, but more for women than for men. Young adults are taking their own lives in alarmingly high numbers, constituting a public health crisis. Suicide ranks first as the cause of death in India in both the age groups of 15–29 years and 15–39 years, as compared with its second and third rank globally in these age groups, respectively. The increasing SDR observed among the elderly in recent years will pose additional challenges. There are large differences in suicide deaths by sex and the SDRs vary substantially between the states. The trends presented over time for the Indian states by sex and age groups can inform suicide prevention policies and monitoring of suicide burden at the state level.

The SDR for women in India was slightly higher in the 1990s than for men, converging in 2001 and then diverging from 2002 with a decrease in the rate for women whereas the rate in men continued to be stagnant. India's men-to-women SDR ratio was lower than the global ratio in 2016. Suicide deaths vary by sex around the world, with SDR in most countries higher in men than in women.^3^ Several theories of convergence and divergence of the men-to-women SDR ratio with modernisation have been tested globally based on the hypothesis that it affects men and women differently with conflicting results.^16^ A previous attempt at understanding this relation for India using administrative data for suicide deaths was inconclusive.^17^ It is also speculated that these gender differences in SDR might be relatively less pronounced if suicide attempts were considered because women make more suicide attempts than men, but men are more likely to die in their attempts than women.^3^, ^18^

The national level estimates mask the large variations seen in suicide deaths at the state level in India, as evident from the wide range of SDR, men-to-women ratio, and observed-to-expected ratio for the states. Overall, suicides accounted for a lower proportion of deaths in the relatively less developed low and lower-middle ETL state groups than in the higher-middle and high ETL state groups. For both sexes, the differences in SDR were quite pronounced when comparing the state-level data that show a geographical divide. The southern states of Andhra Pradesh, Karnataka, Tamil Nadu, and Telangana, which are in the higher-middle and high ETL groups, consistently had a higher SDR for both men and women.^7^, ^16^ The central and western states show mid-level rates, with the exception of Chhattisgarh that had a high SDR among men. SDRs in the north and north-western states were generally low. A mixed pattern was seen for the eastern states, with West Bengal having higher rates than the other states. In the north-eastern states, Tripura had among the highest SDRs and the other states had a mix of high and low SDRs, with the men-to-women SDR ratios generally much higher than the average for India. The levels of urbanisation, proportion of literate population, and difference in literacy attainment between men and women have been suggested as reasons for the variations in suicide deaths at the state level in India.^7^, ^9^, ^16^

The nearly three times higher SDR observed in women in India as compared with the rate expected globally for geographies at similar levels of Socio-Demographic Index highlights the particular need to better understand the determinants of suicides among women in India. Globally and in India, differences in socially acceptable methods of dealing with stress and conflict for women and men, availability of and the preference for different means of suicide, differences in alcohol consumption patterns, domestic violence, poverty, and differences in care-seeking rates for mental disorders between women and men have been cited for gender differentials in SDR.^3^, ^19^, ^20^, ^21^, ^22^, ^23^ Married women account for the highest proportion of suicide deaths among women in India.^7^, ^8^ Marriage is known to be less protective against suicide for women because of arranged and early marriage, young motherhood, low social status, domestic violence, and economic dependence.^3^, ^13^, ^14^, ^15^, ^16^, ^17^, ^18^, ^19^, ^20^, ^21^, ^22^, ^23^, ^24^, ^25^ The trends in SDR in women in this study suggest the need to further assess the complex relationships between gender and suicidal behaviour to facilitate women-specific suicide prevention strategies.^8^, ^22^, ^24^ The Protection of Women from Domestic Violence Act has been in place in India since 2005, and it would be prudent to understand the effect it has had on suicide prevention among married women.^26^ Perhaps, there are lessons to be learnt from China, which had one of the highest female SDRs in 1990 but reduced it by 70% in 2016.^1^, ^27^ Nonetheless, SDR among men in India did not change much from 1990 to 2016, and remains higher than the global average, although not as striking as SDR in women.^1^ One of the reasons for this stagnation among men could be that only suicides of farmers have received attention from policy makers and the media in India, which could have resulted in neglect in dealing with suicide prevention in men overall.^8^, ^9^, ^28^, ^29^ The persistent high suicide rate among men in India needs to be addressed.

A bimodal pattern for suicide deaths was seen for women in our data, with a peak in suicide rates in younger women and then an increase after 70 years of age, which did not vary by ETL group. A somewhat similar pattern emerged for men as well, although the peak in the younger ages was much less distinct compared with that in women. Distinct age-related patterns in suicide death risk at the country level corresponding to different stages of economic development have been reported, with a bimodal pattern—as seen in India—seen in countries at an intermediate stage of industrialisation.^9^, ^30^ One pronounced finding is that despite a reduction in SDR between 1990 and 2016 in younger women, the SDR among them continues to remain high. Recently, high suicide deaths in adolescent girls have gained attention, with suicides having surpassed maternal mortality as the leading cause of death globally.^31^, ^32^ Several forms of gender role differentiation and gender-based discrimination have been highlighted, including early marriage and a higher risk of depression, as possible reasons for this high SDR.^31^, ^33^ India accounted for one-third of the global child marriages in 2014.^34^ India launched its National Programme for Adolescent Health in 2014 that aimed to address mental, sexual, and reproductive health among other health needs.^35^ The programme has various indicators to track age at marriage and teenage pregnancies, depression, and gender-based violence, but does not explicitly mention suicidal ideation as an indicator, tracking of which is imperative given the study findings.^36^ Furthermore, there is a need to better understand the linkages between mental health and sexual and reproductive health, and rights for adolescent girls in India that constrain their aspirations and opportunities leading to higher suicide deaths.^31^, ^32^, ^33^, ^36^ For suicide among men in India, it appears that young adults are a vulnerable group, and marriage does not seem to be protective for them either.^37^ For the elderly, social isolation, depression, functional disability, and the feeling of being a burden on their family have been cited as reasons for suicidal ideation.^38^, ^39^, ^40^ However, not much is known about suicide in the elderly in India. With their increasing proportion in the population over time, the reasons for suicidal ideation and mental health issues in the elderly need to be explored urgently within the National Programme for Health Care of the Elderly in India to address the increasing suicide deaths in this age group.^41^

Personal or social factors such as socioeconomic circumstances, interpersonal, social and cultural conflicts, alcoholism, financial problems, unemployment, and poor health are known as major reasons for suicide in India for both men and women.^8^, ^20^, ^25^, ^42^, ^43^ The use of poison, medication or drug overdose, and hanging have been reported as the most used means of suicide.^7^, ^8^, ^44^, ^45^, ^46^ The reasons for and the means used for suicide highlight the need to address the underlying social determinants of health through macroeconomic policies to protect the vulnerable in order to reduce suicide rates at the population level,^8^, ^47^ to limit or reduce harmful use of pesticides or medicine by relevant regulatory frameworks,^48^, ^49^, ^50^ and to promote access to and availability of mental health services.^51^, ^52^ Until 2017, suicide was a criminal offence in India, which led to under-reporting of suicide deaths in the National Crime Records Bureau of India.^7^, ^8^, ^53^, ^54^ Its decriminalisation is expected to have a major role in access to mental health treatment and possible reduction in under-reporting and stigma associated with suicide.^54^ Furthermore, appropriate reporting of suicides in the media could also contribute to possible reduction in stigma.^55^ The National Mental Health Policy of India launched in 2014, which explicitly aims to reduce suicide deaths and suicide attempts using various strategies;^56^ however, the implemntation of this mental health programme has left much to be desired.^57^ Very little has been done thus far for suicide prevention in India, and the projections for the SDG 2030 target are dismal, with the majority of the states with more than 80% of India's population having less than 10% probability of reaching the SDG target. A comprehensive national suicide prevention strategy that systematically addresses the gender-specific multi-sectoral nature of suicide along with mental health is urgently needed to accelerate the probability of closing the gap towards the SDG target.^3^, ^8^, ^18^, ^58^, ^59^

The general limitations of the GBD methodology and those for injuries including suicide are published elsewhere.^2^, ^10^, ^11^, ^13^ A specific limitation for India is an incomplete medically certified cause of death system that covers only a small proportion of the deaths in India and has variable coverage across the states.^60^ Verbal autopsy data from the Sample Registration System, a major source for cause of death data for the states of India, were useful for the findings in this paper. Verbal autopsy cause of death data are generally considered a reasonable alternative for cause of death distribution at the population level when vital registration data are inadequate, as in India.^10^ However, since verbal autopsy cause of death data are generally unavailable before 2000 in India, the suicide death estimates before this period are driven by covariates. Suicide trends over time reported in this paper are the best possible estimates with the available data. Improvement in the medically certified cause of death system would enable a more robust cause of death understanding in India. Additionally, because of the sensitive nature of suicide and the classification of suicide as a criminal offence until recently in India, under-reporting of suicide deaths is known and misclassification is possible.^3^, ^7^, ^8^ However, the GBD methodology addresses this undercount in suicide deaths by redistribution of improbable causes of deaths to the most likely underlying causes, which is a substantial strength of the findings presented in this report. The use of a variety of available data sources that could be accessed, and the substantial contribution of a network of experts from India in the analysis and interpretation of the findings are other strengths of the findings in this report.

In conclusion, the disproportionately high suicide deaths in India are a public health crisis. Suicide ranks as the leading cause of death among young adults in India, and suicides among women need particular attention. This report provides a comprehensive assessment of the trends of suicide deaths in every state of India over the past quarter century. A national suicide prevention strategy is needed as a guide, which then has to be adapted at the state level to take into account the wide variations in trends between the states and the context of each state to reduce the burden of suicide deaths in India.
