# Supplementary material for: Gender differentials and state variations in suicide deaths in India: the Global Burden of Disease Study 1990–2016
Source: Lancet Public Health. 2018 Sep 12;3(10):e478–89. doi: 10.1016/S2468-2667(18)30138-5 (PMC6178873; doi:10.1016/S2468-2667(18)30138-5)
Supplement: Supplementary appendix [file mmc1.pdf]

THE LANCET
Public Health

## Supplementary appendix

This appendix formed part of the original submission and has been peer reviewed. We post it as supplied by the authors.

Supplement to: India State-Level Disease Burden Initiative Suicide Collaborators. Gender differentials and state variations in suicide deaths in India: the Global Burden of Disease Study, 1990–2016. *Lancet Public Health* 2018; published online Sept 12. http://dx.doi.org/10.1016/S2468-2667(18)30138-5.

# Gender differentials and state variations in suicide deaths in India: the Global Burden of Disease Study 1990-2016

India State-Level Disease Burden Initiative Suicide Collaborators

## Web Appendix

Correspondence to: Prof. Rakhi Dandona, rakhi.dandona@phfi.org

**Table of Contents**


1. GBD 2016 methods for suicide mortality estimation and projection to 2030 ........ 3
2. GBD 2016 India data inputs for suicide deaths and covariates ........ 6
3. GBD 2016 list of covariates used for suicide death estimation ........ 12
4. Age-standardised suicide death rate for women in 20 countries with the highest rates in women, 2016 ...... 13
5. Number of suicide deaths among women and men in the states of India, 2016 ........ 14
6. Suicide death rate among women and men in India by age group, 1990 and 2016 ........ 15
7. Number and percent of suicide deaths by age group in India, 1990 and 2016 ........ 16
8. Percent of total deaths due to suicide in young adults by sex in the states of India, 2016 ........ 17
9. Men-to-women suicide crude death rate ratio by age in India and in the states grouped by epidemiological transition level, 1990 and 2016 ........ 18
10. Men-to-women suicide death rate ratio in the states of India, 1990 to 2016 ........ 19
11. Projected suicide death rate for both sexes combined and the probability of reaching the SDG 2030 target for the states of India ........ 20
12. All-age suicide death rate among women and men in states grouped by epidemiological transition level by age, 1990 and 2016 ........ 21

## 1. GBD 2016 methods for suicide mortality estimation and projection to 2030

The Global Burden of Disease Study (GBD 2016) study estimated mortality due to 264 causes by age, sex, and location between 1990 and 2016 for 195 countries and territories, the detailed methods for which are available in the GBD 2016 cause of death capstone paper [Lancet 2017; 390: 1151–1210]. GBD 2016 also produced projections up to 2030 for health-related SDG indicators based on past trends, the detailed methods for which are available in the GBD 2016 SDG capstone paper [Lancet 2017; 390: 1423–1459]. Here, we provide an overview of the methods used by GBD 2016 for cause of death estimation and projections to 2030, with some details specific to the estimation of fatal outcomes of suicide.

### ICD codes mapped to suicide mortality in GBD

In GBD 2016, suicide was defined following the International Classification of Diseases (ICD) as death caused by purposely self-inflicted poisoning or injury (ICD-10 codes X60-X64.9, X66-X84.9, Y87.0; ICD-9 codes E950-E959); deaths from unintentional drug overdoses are not included in this definition. For this analysis, we present results for suicide as an aggregate cause of death.

### Suicide mortality data sources

A detailed description of the data sources for the cause of death database can be found in the appendix to GBD 2016 cause of death capstone paper [Lancet 2017; 390: 1151–1210]. These sources include vital registration systems and verbal autopsy reports. For India, the major data input to determine suicides mortality in India was the Sample Registration System cause of death data and some other studies. The SRS in India is operated by the Office of the Registrar General of India working under the Ministry of Home Affairs, Government of India. Cause of death data from SRS verbal autopsy covers 455,460 deaths from the rural and urban populations of every state of India from 2004 to 2013 in which physicians assigned the cause of death based on the information provided in the verbal autopsy interview of a person close to each deceased person. Using the 2001 census, 7597 geographic units, 4433 (58·4%) of which were rural, were sampled for the 2004–13 SRS to represent the population of each state and union territory of India, ultimately with a sample of 6·7 million people that was equivalent to 0·7% of India's population. The SRS cause of death data for 2004–06, 2007–09, and 2010–13 were provided for each state and union territory by the Office of the Registrar General of India for use in the state-level disease burden estimation. We used 2005, 2008, and 2012 as midpoint years for these three time periods. The inclusion of SRS 2004–13 data in this analysis offers a comprehensive picture of causes of death in India. In the absence of a fully functional vital registration system, verbal autopsy can provide reasonable population level cause of death distribution. Since the cause of death data are unavailable before the year 2000 in India, the estimation of suicide deaths before this period are mainly driven from covariates. The list of data sources for estimating suicide deaths and for the covariates used is shown in this appendix (pp 6-11).

### Addressing bias in input data

Variation in data quality was addressed in GBD 2016 through a series of methods that include data standardisation and the redistribution of inappropriately coded deaths or "garbage codes" that are not possible causes of death, or that are not specific underlying causes of death but have been entered as the underlying cause of death on death certificates. Undercounting or miss-assignment of deaths from suicide is a known problem in suicide death estimation, and the level and type of miss-assignment differs by location, age, and sex. Correction of miss-assignment is accounted for in GBD in part by reassignment from ICD codes that may include suicide deaths, such as undetermined intent injury codes (Y10-Y34 in ICD-10 and E980-E988 in ICD-9) or exposure to unspecified factor (X59 in ICD-10; E887 in ICD-9), some intermediate causes of death that cannot be specific underlying causes of death (eg septicemia or peritonitis), or as poorly defined or unknown causes of mortality (R99). For distribution of intermediate causes, we used a regression between suicide fractions and intermediate causes by age and sex in each location for each cause of injury. The same regressions were implemented for homicide and unintentional injuries. Based on scale up betas from these three regressions to one, we redistributed deaths coded to indeterminate causes to suicide, homicide, and unintentional injury. Redistribution of garbage codes is explained in the GBD 2016 cause of death capstone paper [Lancet 2017; 390: 1151–210]. Specifically for India, in the Sample Registration System verbal autopsy data, 86.8% of the deaths estimated to be from suicide were directly assigned to suicide in these data, 11.6% were assigned to suicide based on redistribution of garbage codes, and 1.4% assigned to suicide from correction for miss-assignment.

## Suicide mortality estimation

The approach to cause of death estimation for injuries, including suicide, was as shown in this flowchart:

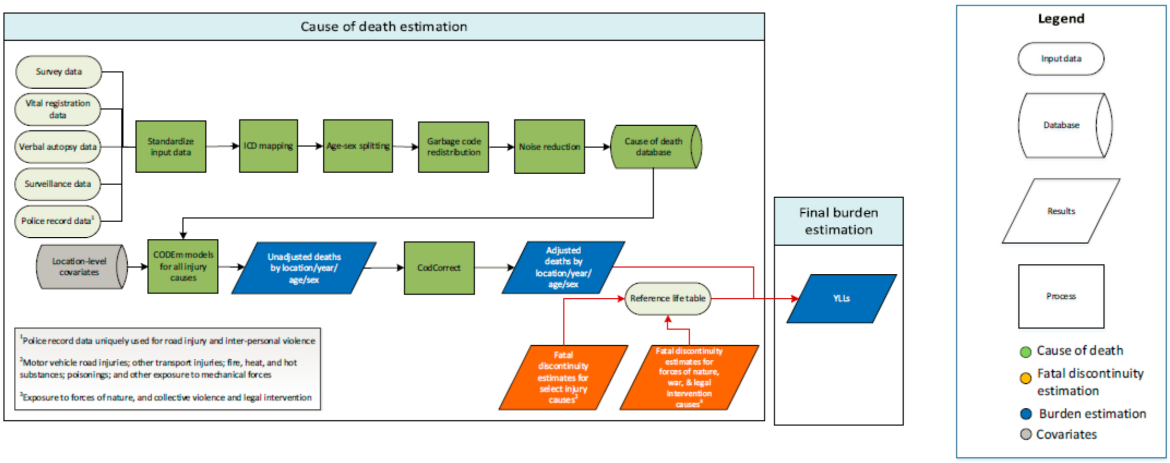


Mortality from suicide was estimated using the cause of death ensemble model (CODEm) developed for the GBD study. Ensemble modelling is a method where a large number of model specifications are systematically tested and reviewed based on their out-of-sample predictive validity; models that perform best are subsequently incorporated into a weighted ensemble model with the highest weights assigned to models with the best out-of-sample prediction error. The model for suicide was age-limited such that deaths from self-harm are restricted to a lower limit of age 10 years, due to the difficulty of determining intent for deaths at younger ages. A description of CODEm follows.

CODEm framework relies on four key components. First, all available data are identified and gathered to be used in the modelling process. Though the data may vary in quality, they all contain some signal of the true epidemiological process. Second, a diverse set of plausible models are developed to capture well-documented associations in the estimates. Using a wide variety of individual models to create an ensemble predictive model has been shown to outperform techniques using only a single model both in cause of death estimation and in more general prediction applications. Third, the out-of-sample predictive validity is assessed for all individual models, which are then ranked for use in the ensemble modelling stage. Finally, differently weighted combinations of individual models are evaluated to select the ensemble model with the highest out-of-sample predictive validity.

As many factors covary with a particular cause of death, a large range of plausible statistical models are developed for each cause. For the CODEm framework, four families of statistical models are developed using covariates. These are mixed effects linear models of the natural log of the death rate, mixed effects linear models of the logit of the cause fraction, spatiotemporal Gaussian process regression (ST-GPR) models of the log of the death rate, and ST-GPR of the logit of the cause fraction. All plausible relationships between covariates and relevant cause are identified, and all possible permutations of selected covariates are tested in linear models where the logit cause fraction or log death rate is the response variable. Because we test all permutations of covariates, multicollinearity between covariates may produce implausible signs on coefficients or unstable coefficients. All models where the sign on the coefficient is in the direction expected based on the literature and where the coefficient is statistically significant at $p < 0.05$ are retained. We run covariate selection for both cause fractions and death rates and then create both mixed effects only and ST models for each set of covariates.

The performance of all component models and ensembles is evaluated using out-of-sample predictive validity tests. Thirty percent of the data are excluded from the initial model fits, and half of that (15% of total) is used to evaluate and rank component models and then build ensembles. Data are held out from the analysis using the pattern of missingness for each cause in the cause of death database. Out-of-sample predictive validity testing is repeated until stable model results have been obtained. The out-of-sample performance tests include the root mean squared error (RMSE) of the log of the cause-specific death rate, the direction of the trend in the prediction compared to the data, and the validity of the 95% uncertainty interval (UI). For every model, we show the in-sample root mean squared error of the log death rates (RMSE) and the out-of-sample performance in the 15% of data not used in the model building process. After component models are ranked on their out-of-sample

predictive validity they are weighted based on their ranking and each component model contributes a portion to the final estimate. How much each submodel contributes is a function of its relative ranking as well as the value of psi chosen, which dictates that distribution of rankings. Using the second half of the holdout data (15% of total), the differently weighted ensembles and different values of psi are tested using the same predictive validity metrics as the component models. For every model, we show the in-sample RMSE of the log death rates and the out-of-sample performance in the 15% of data not used in the model building process. The ensemble with the best average trend and RMSE is chosen as the final ensemble weighting scheme. After a model weighting scheme has been chosen, each model contributes a number of draws proportional to its weight such that 1,000 draws are created. The mean of the draws is used as the final estimate for the CODEm process and 95% UI are created from the 0.025 and 0.975 quantiles of the draws. The final assessment of ensemble model performance is the validity of the UIs; ideally, the 95% UI for a model would capture 95% of the data out-of-sample. Higher coverage suggests that UIs are too large and lower than 95% suggest UIs are too narrow. Separate models were run for male and female suicide mortality, and the age range for both models was 10 to 95+ years.

CODEm models estimate the individual cause-level mortality without taking into account the all-cause mortality. GBD uses the CodCorrect algorithm to ensure that all individual causes add up to the all-cause mortality. After generating underlying cause of death estimates and accompanying uncertainty, this algorithm combines these models into estimates that are consistent with the levels of all-cause mortality estimated for each age-sex-year-location group. Using 1000 draws from the posterior distribution of each cause and 1000 draws from the posterior distribution of the estimation of all-cause mortality, CoDCorrect rescales the sum of cause-specific estimates to equal the draws from the all cause distribution.

**Projection of suicide death rate to 2030**

GBD 2016 produced projections for the health-related SDG indicators up to 2030 based on past trends. The steps for estimating the projected annual rate of change for age-standardised suicide death rate (SDR) from 2017 to 2030 for each location were:

- Annual percent rate of change of SDR was calculated from 1990 to 2016 for each year from the pervious year. This was converted to natural log, as this is more suitable than using the direct annual percent change to compute projections.
- The weight for each year was calculated using this formula:

  $$weight_{year} = \frac{(\text{year} - 1990)^{\omega}}{\sum_{t=1991}^{T} (t - 1990)^{\omega}}$$

  where ω is the weight function the value of which denotes how much higher impact recent years would have compared with the past years when calculating the annual rate of change for the projection. To determine the appropriate value of ω for each indicator, an out-of-sample predictive validity test was done using data from 1990 to 2007 to predicted values for the years from 2008 to 2016. Assuming a range of values from 0 to 2 for *ω* with an increments of 0.2, the best predicted value for the period of 2008 to 2016 was tested for each indicator. The final value for the weight function (ω) specific to each indicator for projection was chosen that minimised the root mean squared error in the 2008–16 projections based on the 1990-2007 data. With this approach, the weight function (ω) for SDR was determined as 2, which was used to calculate the weight for each year from 1991 to 2016 with the formula shown above. To illustrate the difference in the influence of the recent versus past trends of SDR, if the weight for the rate of change from 2015 to 2016 were 1, the weights for the rates of change 5, 10, 15 and 20 years ago would be 0.65, 0.38, 0.18 and 0.05, respectively.
- The natural log of the rate of change for each year calculated above was multiplied by the weight for each year to arrive at the weighted natural log rate of change for each year. This was summed for all years from 1990 to 2016, and the inverse of this sum of the natural log rates of change gave the weighted annual rate of change to be used for the projections from 2017 to 2030.

**Uncertainty intervals**

GBD estimates uncertainty intervals for all estimates. Point estimates for each quantity of interest were derived from the mean of the draws, while 95% uncertainty intervals (UIs) were derived from the 2.5$^{th}$ and 97.5$^{th}$ percentiles of the 1000 draw level values. Uncertainty in the estimation is attributable to sample size variability within data sources, different availability of data by age, sex, year, or location, and cause specific model specifications. We determined UIs for components of cause-specific estimation based on 1000 draws from the posterior distribution of cause specific mortality by age, sex, and location for each year included in the GBD 2016 analysis. With this approach, uncertainty could be quantified and propagated into the final quantities of interest.

## 2. GBD 2016 India data inputs for suicide deaths and covariates


| |
|---|
| Aaron R, Joseph A, Abraham S, Muliyil J, George K, Prasad J, Minz S, Abraham VJ, Bose A. Suicides in young people in rural southern India. Lancet. 2004; 363(9415): 1117-8. |
| Ahmad A, Khalique N, Khan Z, Amir A. Prevalence of psychosocial problems among school going male adolescents. Indian J Community Med. 2007; 32(3): 219-21. |
| Bansal PD, Barman R. Psychopathology of school going children in the age group of 10-15 years. Int J Appl Basic Med Res. 2011; 1(1): 43-7. |
| Behera P, Sharan P, Mishra AK, Nongkynrih B, Kant S, Gupta SK. Prevalence and determinants of depression among elderly persons in a rural community from northern India. Natl Med J India. 2016; 29(3): 129–35. |
| Department of Women and Child Development, Ministry of Human Resource Development, Government of India, United Nations Children's Fund (UNICEF) - India Country Office. India Summary Report on the Multiple Indicator Cluster Survey 2000. New Delhi, India: UNICEF India Country Office; 2000. |
| Sonalde D, Vanneman R, National Council of Applied Economic Research, University of Michigan. India Human Development Survey 2005. Ann Arbor, Michigan: Inter-University Consortium for Political and Social Research. |
| Directorate of Economics & Statistics and Office of Chief Registrar (Births & Deaths), Government of National Capital Territory of Delhi. Report on Medical Certification of Cause of Deaths in Delhi-2011. New Delhi, India: Directorate of Economics & Statistics and Office of Chief Registrar (Births & Deaths); 2012. |
| Global Burden of Disease Health Financing Collaborator Network, Institute for Health Metrics and Evaluation (IHME). Global Development Assistance for Health, Government, Prepaid Private, and Out-of-Pocket Health Spending 1995-2014. Seattle, United States: IHME; 2017. |
| Guerra M, Prina AM, Ferri CP, Acosta D, Gallardo S, Huang Y, Jacob KS, Jimenez-Velazquez IZ, Llibre Rodriguez JJ, Liu Z, Salas A, Sosa AL, Williams JD, Uwakwe R, Prince M. A comparative cross-cultural study of the prevalence of late life depression in low and middle income countries. J Affect Disord. 2016; 190: 362–8. |
| INDEPTH Network. Africa, Asia, Oceania - INDEPTH Network Cause-Specific Mortality - Release 2014. Accra, Ghana: INDEPTH Network; 2014. |
| Indian Council of Medical Research (ICMR). India Study on Causes of Death by Verbal Autopsy 2003. New Delhi, India: ICMR. [Data shared for this analysis] |
| Indian Council of Medical Research, World Health Organization. India STEPS Non communicable Disease Risk Factors Survey 2003-2005. [Data shared for this analysis] |
| Institute of Health Systems, World Health Organization (WHO). WHO Multi-country Survey Study Report on Health and Health System Responsiveness, Andhra Pradesh 2000-2001. |
| International Institute for Population Sciences (IIPS), Ministry of Health and Family Welfare, Government of India, Macro International. India National Family Health Survey Data (NFHS-1) 1992-1993. Mumbai, India: IIPS. |
| International Institute for Population Sciences (IIPS), Ministry of Health and Family Welfare, Government of India, Macro International. India National Family Health Survey (NFHS-1) 1992-1993: National Report. Mumbai, India: IIPS. |
| International Institute for Population Sciences (IIPS), Ministry of Health and Family Welfare, Government of India, ORC Macro. India National Family Health Survey Data (NFHS-2) 1998-1999. Mumbai, India: IIPS. |
| International Institute for Population Sciences (IIPS), Ministry of Health and Family Welfare, Government of India, ORC Macro. India National Family Health Survey (NFHS-2) 1998-1999: National and State Reports. Mumbai, India: IIPS. |
| International Institute for Population Sciences (IIPS), Ministry of Health and Family Welfare, Government of India, Macro International. India National Family Health Survey Data (NFHS-3) 2005-2006. Mumbai, India: IIPS. |
| International Institute for Population Sciences (IIPS), Ministry of Health and Family Welfare, Government of India, Macro International. India National Family Health Survey (NFHS-3) 2005-2006: National and State Reports. Mumbai, India: IIPS. |

International Institute for Population Sciences (IIPS), Ministry of Health and Family Welfare, Government of India, ICF. India National Family Health Survey (NFHS-4) 2015-2016: National and State-level Factsheets. Mumbai, India: IIPS.

International Institute for Population Sciences (IIPS), Ministry of Health and Family Welfare, Government of India. India District Level Household Survey Data (DLHS-1) 1998-1999. Mumbai, India: IIPS.

International Institute for Population Sciences (IIPS), Ministry of Health and Family Welfare, Government of India. India District Level Household Survey (DLHS-1) 1998-1999: National Report. Mumbai, India: IIPS.

International Institute for Population Sciences (IIPS), Ministry of Health and Family Welfare, Government of India. India District Level Household Survey Data (DLHS-2) 2002-2004. Mumbai, India: IIPS.

International Institute for Population Sciences (IIPS), Ministry of Health and Family Welfare, Government of India. India District Level Household Survey (DLHS-2) 2002-2004: National and State Reports. Mumbai, India: IIPS.

International Institute for Population Sciences (IIPS), Ministry of Health and Family Welfare, Government of India. India District Level Household and Facility Survey Data (DLHS-3) 2007-2008. Mumbai, India: IIPS.

International Institute for Population Sciences (IIPS), Ministry of Health and Family Welfare, Government of India. India District Level Household and Facility Survey (DLHS-3) 2007-2008: National and State Reports. Mumbai, India: IIPS.

International Institute for Population Sciences, Ministry of Health and Family Welfare, Government of India. India District Level Household and Facility Survey Data (DLHS-4) 2012-2013. Mumbai, India: IIPS.

International Institute for Population Sciences (IIPS), Ministry of Health and Family Welfare, Government of India. India District Level Household and Facility Survey Data (DLHS-4) 2012-2013: State Reports. Mumbai, India: IIPS.

International Institute for Population Sciences, World Health Organization (WHO). India WHO Study on Global Ageing and Adult Health 2007-2008.

International Institute for Population Sciences, World Health Organization. India World Health Survey 2003.

Joshi R, Cardona M, Iyengar S, Sukumar A, Raju CR, Raju KR, Raju K, Reddy KS, Lopez A, Neal B. Chronic diseases now a leading cause of death in rural India – mortality data from the Andhra Pradesh Rural Health Initiative. Int J Epidemiol. 2006; 35(6): 1522-9.

Joshi R, Guggilla R, Praveen D, Maulik PK. Suicide deaths in rural Andhra Pradesh--a cause for global health action. Trop Med Int Health. 2015; 20(2): 188–93.

Kalasapati L, Ivaturi S, Reddy P, Babu S. Incidence of suicides in three villages of Khammam district of South India. AP J Psychol Med. 2014; 15(1): 103-7.

Kessler RC, Birnbaum HG, Shahly V, Bromet E, Hwang I, McLaughlin KA, Sampson N, Andrade LH, de Girolamo G, Demyttenaere K, Haro JM, Karam AN, Kostyuchenko S, Kovess V, Lara C, Levinson D, Matschinger H, Nakane Y, Browne MO, Ormel J, Posada-Villa J, Sagar R, Stein DJ. Age differences in the prevalence and co-morbidity of DSM-IV major depressive episodes: results from the WHO World Mental Health Survey Initiative. Depress Anxiety. 2010; 27(4): 351–64.

Kumar R, Sharma AK, Barik S, Kumar V. Maternal mortality inquiry in a rural community of north India. Int J Gynaecol Obstet. 1989; 29(4): 313-9.

Mathias K, Goicolea I, Kermode M, Singh L, Shidhaye R, Sebastian MS. Cross-sectional study of depression and help-seeking in Uttarakhand, North India. BMJ Open. 2015; 5(11): e008992.

Ministry of Health and Family Welfare, Government of India, National Institute of Mental Health and Neurosciences, World Health Organization. India National Mental Health Survey Data Tables 2015-2016. [Data shared for this analysis]

Ministry of Health and Family Welfare, Government of India, National Institute of Mental Health and Neurosciences, World Health Organization. India National Mental Health Survey Report 2015-2016. New Delhi, India: Ministry of Health and Family Welfare; 2016.

Ministry of Health and Family Welfare, Government of India, United Nations Children's Fund. India Coverage Evaluation Survey 2005.

| |
|---|
| Ministry of Health and Family Welfare, Government of India, United Nations Children's Fund. India Coverage Evaluation Survey 2007. |
| Ministry of Health and Family Welfare, Government of India, United Nations Children's Fund. India Coverage Evaluation Survey Data 2009-2010. [Data shared for this analysis] |
| Ministry of Statistics and Programme Implementation, Government of India. Consumer Expenditure Survey, National Sample Survey Round 38, January-December 1983. New Delhi, India: Ministry of Statistics and Programme Implementation. |
| Ministry of Statistics and Programme Implementation, Government of India. Survey on Participation in Education, National Sample Survey Round 42, July 1986- June 1987. New Delhi, India: Ministry of Statistics and Programme Implementation. |
| Ministry of Statistics and Programme Implementation, Government of India. Consumer Expenditure Survey, National Sample Survey Round 43, July 1987-June 1988. New Delhi, India: Ministry of Statistics and Programme Implementation. |
| Ministry of Statistics and Programme Implementation, Government of India. Consumer Expenditure Survey, National Sample Survey Round 47, July - December 1991. New Delhi, India: Ministry of Statistics and Programme Implementation. |
| Ministry of Statistics and Programme Implementation, Government of India. Survey on Literacy and Culture, National Sample Survey Round 47, July - December 1991. New Delhi, India: Ministry of Statistics and Programme Implementation. |
| Ministry of Statistics and Programme Implementation, Government of India. Consumer Expenditure Survey, National Sample Survey Round 50, July 1993-June 1994. New Delhi, India: Ministry of Statistics and Programme Implementation. |
| Ministry of Statistics and Programme Implementation, Government of India. Employment and Unemployment Survey, National Sample Survey Round 50, July 1993- June 1994. New Delhi, India: Ministry of Statistics and Programme Implementation. |
| Ministry of Statistics and Programme Implementation, Government of India. Consumer Expenditure Survey, National Sample Survey Round 52, July 1995-June 1996. New Delhi, India: Ministry of Statistics and Programme Implementation. |
| Ministry of Statistics and Programme Implementation, Government of India. Participation in Education Survey, National Sample Survey Round 52, July 1995-June 1996. New Delhi, India: Ministry of Statistics and Programme Implementation. |
| Ministry of Statistics and Programme Implementation, Government of India. Survey on Healthcare, National Sample Survey Round 52, July 1995-June 1996. New Delhi, India: Ministry of Statistics and Programme Implementation. |
| Ministry of Statistics and Programme Implementation, Government of India. Consumer Expenditure Survey, India National Sample Survey Round 55, July 1999-June 2000. New Delhi, India: Ministry of Statistics and Programme Implementation. |
| Ministry of Statistics and Programme Implementation, Government of India. Employment and Unemployment Survey, India National Sample Survey Round 55, July 1999-June 2000. New Delhi, India: Ministry of Statistics and Programme Implementation. |
| Ministry of Statistics and Programme Implementation, Government of India. Consumer Expenditure Survey, National Sample Survey Round 60, January-June 2004. New Delhi, India: Ministry of Statistics and Programme Implementation. |
| Ministry of Statistics and Programme Implementation, Government of India. Employment and Unemployment surveys, National Sample Survey Round 60, January-June 2004. New Delhi, India: Ministry of Statistics and Programme Implementation. |
| Ministry of Statistics and Programme Implementation, Government of India. Survey on Morbidity and Healthcare, National Sample Survey Round 60, January-June 2004. New Delhi, India: Ministry of Statistics and Programme Implementation. |
| Ministry of Statistics and Programme Implementation, Government of India. Consumer Expenditure Survey, National Sample Survey Round 61, July 2004 - June 2005. New Delhi, India: Ministry of Statistics and Programme Implementation. |
| Ministry of Statistics and Programme Implementation, Government of India. Employment and Unemployment Survey, National Sample Survey Round 61, July 2004- June 2005. New Delhi, India: Ministry of Statistics and Programme Implementation. |
| Ministry of Statistics and Programme Implementation, Government of India. Consumer Expenditure Survey, National Sample Survey Round 64, July 2007- June 2008. New Delhi, India: Ministry of Statistics and Programme Implementation. |

Ministry of Statistics and Programme Implementation, Government of India. Employment and Unemployment Survey, National Sample Survey Round 64, July 2007- June 2008. New Delhi, India: Ministry of Statistics and Programme Implementation.

Ministry of Statistics and Programme Implementation, Government of India. Participation & Expenditure in Education Survey, National Sample Survey Round 64, July 2007- June 2008. New Delhi, India: Ministry of Statistics and Programme Implementation.

Ministry of Statistics and Programme Implementation, Government of India. Social Consumption Survey on Health and Education, National Sample Survey Round 71, January 2014 - June 2014. New Delhi, India: Ministry of Statistics and Programme Implementation.

Ministry of Statistics and Programme Implementation, Government of India. Household expenditure on services and durable goods survey, National Sample Survey Round 72, July 2014 - June 2015. New Delhi, India: Ministry of Statistics and Programme Implementation.

Ministry of Statistics and Programme Implementation, Government of India. India State Series: Gross State Domestic Product at Factor Cost by Industry of Origin Tables 1980-81 to 1993-94. New Delhi, India: Ministry of Statistics and Programme Implementation.

Ministry of Statistics and Programme Implementation, Government of India. India State Series: Gross State Domestic Product at Factor Cost by Industry of Origin Tables 1993-94 to 2004-05. New Delhi, India: Ministry of Statistics and Programme Implementation.

Ministry of Statistics and Programme Implementation, Government of India. India State Series: Gross State Domestic Product at Factor Cost by Industry of Origin Tables 1999-2000 to 2009-10. New Delhi, India: Ministry of Statistics and Programme Implementation.

Ministry of Statistics and Programme Implementation, Government of India. India State Series: Gross State Domestic Product at Factor Cost by Industry of Origin Tables 2004-05 to 2013-14. New Delhi, India: Ministry of Statistics and Programme Implementation.

National Institute of Medical Statistics, Indian Council of Medical Research (ICMR), Integrated Disease Surveillance Programme. Non-Communicable Disease Risk Factors Survey Data 2007-2008. New Delhi, India: ICMR. [Data shared for this analysis]

National Institute of Medical Statistics, Indian Council of Medical Research (ICMR), Integrated Disease Surveillance Programme. Non-Communicable Disease Risk Factors Survey Report 2007-2008. New Delhi, India: ICMR.

National Nutrition Monitoring Bureau, National Institute of Nutrition (NIN), Indian Council of Medical Research. India Rural Third Repeat Survey of Diet and Nutritional Status Data 2011-2012. [Data shared for this analysis]

National Nutrition Monitoring Bureau, National Institute of Nutrition (NIN), Indian Council of Medical Research. India Rural Third Repeat Survey of Diet and Nutritional Status Report 2011-2012. Hyderabad, India: NIN.

Office of the Registrar General & Census Commissioner, Ministry of Home Affairs, Government of India. India Annual Health Survey Data 2010-2011. New Delhi, India: Office of the Registrar General & Census Commissioner.

Office of the Registrar General & Census Commissioner, Ministry of Home Affairs, Government of India. India Annual Health Survey Report 2010-2011. New Delhi, India: Office of the Registrar General & Census Commissioner.

Office of the Registrar General & Census Commissioner, Ministry of Home Affairs, Government of India. India Annual Health Survey Data 2011-2012. New Delhi, India: Office of the Registrar General & Census Commissioner.

Office of the Registrar General & Census Commissioner, Ministry of Home Affairs, Government of India. India Annual Health Survey Report 2011-2012. New Delhi, India: Office of the Registrar General & Census Commissioner.

Office of the Registrar General & Census Commissioner, Ministry of Home Affairs, Government of India. India Annual Health Survey Data 2012-2013. New Delhi, India: Office of the Registrar General & Census Commissioner.

Office of the Registrar General & Census Commissioner, Ministry of Home Affairs, Government of India. India Annual Health Survey Report 2012-2013. New Delhi, India: Office of the Registrar General & Census Commissioner.

Office of the Registrar General & Census Commissioner, Ministry of Home Affairs, Government of India. India Medical Certification of Cause of Death Report 2008. New Delhi, India: Office of the Registrar General & Census Commissioner.

Office of the Registrar General & Census Commissioner, Ministry of Home Affairs, Government of India. India Population and Housing Census 1981. New Delhi, India: Office of the Registrar General & Census Commissioner.

| |
|---|
| Office of the Registrar General & Census Commissioner, Ministry of Home Affairs, Government of India. India Population and Housing Census 2001. New Delhi, India: Office of the Registrar General & Census Commissioner. |
| Office of the Registrar General & Census Commissioner, Ministry of Home Affairs, Government of India. India SRS Statistical Report 1999. New Delhi, India: Office of the Registrar General & Census Commissioner. |
| Office of the Registrar General & Census Commissioner, Ministry of Home Affairs, Government of India. India SRS Statistical Report 2001. New Delhi, India: Office of the Registrar General & Census Commissioner; 2005. |
| Office of the Registrar General & Census Commissioner, Ministry of Home Affairs, Government of India. India SRS Statistical Report 2002. New Delhi, India: Office of the Registrar General & Census Commissioner; 2005. |
| Office of the Registrar General & Census Commissioner, Ministry of Home Affairs, Government of India. India SRS Statistical Report 2003. New Delhi, India: Office of the Registrar General and Census Commissioner; 2005. |
| Office of the Registrar General & Census Commissioner, Ministry of Home Affairs, Government of India. India SRS Statistical Report 2004. New Delhi, India: Office of the Registrar General and Census Commissioner; 2006. |
| Office of the Registrar General & Census Commissioner, Ministry of Home Affairs, Government of India. India SRS Statistical Report 2005. New Delhi, India: Office of the Registrar General and Census Commissioner; 2006. |
| Office of the Registrar General & Census Commissioner, Ministry of Home Affairs, Government of India. India SRS Statistical Report 2007. New Delhi, India: Office of the Registrar General and Census Commissioner; 2008. |
| Office of the Registrar General & Census Commissioner, Ministry of Home Affairs, Government of India. India SRS Statistical Report 2008. New Delhi, India: Office of the Registrar General and Census Commissioner; 2010. |
| Office of the Registrar General & Census Commissioner, Ministry of Home Affairs, Government of India. India SRS Statistical Report 2009. New Delhi, India: Office of the Registrar General and Census Commissioner. |
| Office of the Registrar General & Census Commissioner, Ministry of Home Affairs, Government of India. India SRS Statistical Report 2010. New Delhi, India: Office of the Registrar General and Census Commissioner; 2012. |
| Office of the Registrar General & Census Commissioner, Ministry of Home Affairs, Government of India. India SRS Statistical Report 2011. New Delhi, India: Office of the Registrar General and Census Commissioner; 2013. |
| Office of the Registrar General & Census Commissioner, Ministry of Home Affairs, Government of India. India SRS Statistical Report 2012. New Delhi, India: Office of the Registrar General and Census Commissioner; 2013. |
| Office of the Registrar General & Census Commissioner, Ministry of Home Affairs, Government of India. India SRS Statistical Report 2013. New Delhi, India: Office of the Registrar General and Census Commissioner; 2014. |
| Office of the Registrar General & Census Commissioner, Ministry of Home Affairs, Government of India. India SRS Statistical Report 2014. New Delhi, India: Office of the Registrar General and Census Commissioner. |
| Office of the Registrar General & Census Commissioner, Ministry of Home Affairs, Government of India. India SRS Verbal Autopsy 2004-2006. New Delhi, India: Office of the Registrar General and Census Commissioner. [Data shared for this analysis] |
| Office of the Registrar General & Census Commissioner, Ministry of Home Affairs, Government of India. India SRS Verbal Autopsy Report 2004-2006. New Delhi, India: Office of the Registrar General and Census Commissioner. |
| Office of the Registrar General & Census Commissioner, Ministry of Home Affairs, Government of India. India SRS Verbal Autopsy 2007-2009. New Delhi, India: Office of the Registrar General and Census Commissioner. [Data shared for this analysis] |
| Office of the Registrar General & Census Commissioner, Ministry of Home Affairs, Government of India. India SRS Verbal Autopsy Report 2007-2009. New Delhi, India: Office of the Registrar General and Census Commissioner. |
| Office of the Registrar General & Census Commissioner, Ministry of Home Affairs, Government of India. India SRS Verbal Autopsy 2010-2013. New Delhi, India: Office of the Registrar General and Census Commissioner. [Data shared for this analysis] |

| |
|---|
| Office of the Registrar General & Census Commissioner, Ministry of Home Affairs, Government of India. India SRS Verbal Autopsy Report 2010-2013. New Delhi, India: Office of the Registrar General and Census Commissioner. |
| Patil RN, Nagaonkar SN, Shah NB, Bhat TS. A Cross-sectional Study of Common Psychiatric Morbidity in Children Aged 5 to 14 Years in an Urban Slum. J Fam Med Prim Care. 2013; 2(2): 164-8. |
| Poongothai S, Pradeepa R, Ganesan A, Mohan V. Prevalence of depression in a large urban South Indian population – the Chennai Urban Rural Epidemiology Study (CURES-70). PLoS One. 2009; 4(9): e7185. |
| Public Health Foundation of India. India Cause of Death Estimation Study in Bihar 2011-2014. New Delhi, India: Public Health Foundation of India. [Data shared for this analysis] |
| Rajkumar AP, Thangadurai P, Senthilkumar P, Gayathri K, Prince M, Jacob KS. Nature, prevalence and factors associated with depression among the elderly in a rural south Indian community. Int Psychogeriatr. 2009; 21(2): 372-8. |
| Salve H, Kumar R, Sinha S, Krishnan A. Suicide an emerging public health problem: evidence from rural Haryana, India. Indian J Public Health. 2013; 57(1): 40-2. |
| Sarkar S, Sinha VK, Praharaj SK. Depressive disorders in school children of suburban India: an epidemiological study. Soc Psychiatry Psychiatr Epidemiol. 2012; 47(5): 783-8. |
| Sathyanarayana Rao TS, Darshan MS, Tandon A, Raman R, Karthik KN, Saraswathi N, Das K, Harsha GT, Krishna VST, Ashok NC. Suttur study: An epidemiological study of psychiatric disorders in south Indian rural population. Indian J Psychiatry. 2014; 56(3): 238–45. |
| Shidhaye R, Gangale S, Patel V. Prevalence and treatment coverage for depression: a population-based survey in Vidarbha, India. Soc Psychiatry Psychiatr Epidemiol. 2016; 51(7): 993–1003. |
| Silvanus V, Subramanian P. Epidemiological study of mental morbidity in an urban slum community in India for the development of a community mental health programme. Nepal Med Coll J. 2012; 14(1): 13-7. |
| Srinath S, Girimaji SC, Gururaj G, Seshadri S, Subbakrishna DK, Bhola P, Kumar N. Epidemiological study of child and adolescent psychiatric disorders in urban and rural areas of Bangalore, India. Indian J Med Res. 2005; 122(1): 67-79. |
| United Nations Office on Drugs and Crime (UNODC). World Drug Report 2012. Vienna, Austria: UNODC; 2012. |
| World Health Organization. Study on Global Ageing and Adult Health (SAGE) Pilot Study 2005 Data from the Data Archive of Social Research on Aging. Los Altos, United States: Sociometrics Corporation. Available from: http://home.socio.com/age2728.php |

## 3. GBD 2016 list of covariates used for suicide death estimation

| Covariate | Definition | Direction |
|---|---|---|
| Socio-demographic Index | A measure of development estimated via principal component analysis using log-transformed LDI, TFR, and education years per capita over age 15 years | 0 |
| Log-transformed LDI (I$ per capita) | Lag distributed income per capita (I$): gross domestic product per capita that has been smoothed over the preceding 10 years, log-transformed | 0 |
| Education (years per capita) | Mean level of educational attainment | 0 |
| Population density (500-1000 ppl/sqkm, proportion) | Proportion of the country with population density between 500 and 1000 people per square kilometre | 0 |
| Population density (150-300 ppl/sqkm, proportion) | Proportion of the country with population density between 150 and 300 people per square kilometre | 0 |
| Population density (300-500 ppl/sqkm, proportion) | Proportion of the country with population density between 300 and 500 people per square kilometre | 0 |
| Population density (under 150 ppl/sqkm, proportion) | Proportion of the country with population density under 150 people per square kilometre | 0 |
| Population density (over 1000 ppl/sqkm, proportion) | Proportion of the country with population density over 1000 people per square kilometre | 0 |
| Religion (country indicator) | Binary indicator: country is greater than 50% Muslim | -1 |
| Healthcare access and quality index | Healthcare access and quality index | -1 |
| Major depressive disorder | Major depressive disorder from DISMOD interpolated to be used as covariate | 1 |
| Alcohol (liters per capita) | Domestic supply quantity (litres) of alcohol per adult age 15 years or older per year | 1 |
| Log-transformed SEV scalar: self-harm | All risk factors SEV scalar for the cause self-harm, log-transformed | 1 |

**Source:** GBD 2016 Causes of Death Collaborators. Global, regional, and national age-sex specific mortality for 264 causes of death, 1980-2016: a systematic analysis for the Global Burden of Disease Study 2016. Lancet 2017; 390: 1151-210.

## 4. Age-standardised suicide death rate for women in 20 countries with the highest rates in women, 2016

| Countries | Age-standardised suicide death rate per 100,000 (95% uncertainty interval) |
|---|---|
| Greenland | 38.1 (28.3 to 49.2) |
| Lesotho | 35.3 (16.8 to 54.7) |
| Uganda | 18.7 (12.7 to 25.5) |
| Liberia | 17.0 (12.5 to 21.5) |
| South Korea | 15.5 (10.0 to 22.7) |
| India | 14.7 (13.1 to 16.2) |
| Zimbabwe | 14.0 (9.7 to 18.7) |
| Guyana | 13.1 (10.7 to 15.7) |
| Suriname | 11.3 (9.5 to 13.1) |
| Cameroon | 10.7 (7.1 to 15.0) |
| Solomon Islands | 10.0 (5.8 to 16.1) |
| Japan | 9.7 (8.9 to 10.6) |
| Russia | 9.6 (5.6 to 15.3) |
| Sri Lanka | 9.4 (6.6 to 12.6) |
| Guinea-Bissau | 9.1 (6.2 to 12.6) |
| North Korea | 8.9 (6.3 to 11.9) |
| Federated States of Micronesia | 8.9 (5.4 to 13.5) |
| Chad | 8.9 (5.2 to 13.0) |
| Lithuania | 8.7 (7.6 to 10.0) |
| Togo | 8.7 (6.6 to 11.0) |

**Source**: Institute for Health Metrics and Evaluation. GBD Compare Data Visualization. http://vizhub.healthdata.org/gbd-compare. (Accessed 31 March 2018)

## 5. Number of suicide deaths among women and men in the states of India, 2016

| States of India* | Number of suicide deaths (95% uncertainty interval) | |
|---|---|---|
| | Women | Men |
| **India** | **94,380 (84,002 to 104,274)** | **135,934 (94,305 to 151,239)** |
| Bihar | 3,260 (2,532 to 4,131) | 4,888 (3,744 to 7,385) |
| Jharkhand | 1,306 (911 to 1,733) | 1,759 (1,293 to 2,991) |
| Uttar Pradesh | 14,361 (11,333 to 18,257) | 17,573 (13,793 to 21,464) |
| Rajasthan | 3,006 (2,406 to 3,750) | 5,977 (4,726 to 7,694) |
| Meghalaya | 53 (40 to 67) | 194 (153 to 283) |
| Assam | 2,348 (1,774 to 3,084) | 3,372 (2,602 to 4,454) |
| Chhattisgarh | 1,618 (1,226 to 2,095) | 3,874 (2,410 to 4,934) |
| Madhya Pradesh | 5,087 (3,920 to 6,439) | 8,810 (6,459 to 10,892) |
| Odisha | 2,946 (2,242 to 3,793) | 3,927 (3,031 to 5,469) |
| Arunachal Pradesh | 95 (72 to 126) | 139 (100 to 177) |
| Mizoram | 15 (11 to 20) | 66 (49 to 108) |
| Nagaland | 31 (23 to 42) | 85 (57 to 219) |
| Uttarakhand | 618 (471 to 800) | 747 (567 to 1,036) |
| Gujarat | 5,130 (4,254 to 6,198) | 6,052 (4,813 to 7,297) |
| Tripura | 502 (390 to 632) | 961 (388 to 1,261) |
| Sikkim | 26 (20 to 35) | 55 (42 to 77) |
| Manipur | 142 (110 to 184) | 278 (196 to 354) |
| Haryana | 1,399 (1,115 to 1,750) | 3,052 (2,361 to 3,688) |
| Delhi | 522 (388 to 682) | 995 (722 to 1,575) |
| Telangana | 3,646 (2,808 to 4,596) | 4,676 (2,329 to 6,204) |
| Andhra Pradesh | 5,400 (4,338 to 6,653) | 7,618 (3,750 to 9,575) |
| Jammu and Kashmir | 512 (393 to 677) | 569 (418 to 1,088) |
| Karnataka | 8,147 (6,544 to 10,111) | 12,167 (5,394 to 15,657) |
| West Bengal | 11,088 (8,907 to 13,542) | 13,120 (6,492 to 16,656) |
| Maharashtra | 8,652 (7,022 to 10,482) | 14,241 (9,533 to 17,371) |
| UTs other than Delhi† | 331 (252 to 426) | 493 (308 to 630) |
| Himachal Pradesh | 320 (253 to 403) | 612 (475 to 778) |
| Punjab | 1,086 (845 to 1,361) | 1,768 (1,324 to 3,014) |
| Tamil Nadu | 10,110 (8,303 to 12,270) | 12,510 (5,596 to 16,082) |
| Goa | 81 (70 to 98) | 95 (76 to 120) |
| Kerala | 2,542 (2,189 to 2,956) | 5,260 (1,805 to 6,761) |

*The states are listed in increasing order of epidemiological transition level in 2016.
†Union territories.

## 6. Suicide death rate among women and men in India by age group, 1990 and 2016

| Age group (years) | Women | | | Men | | |
|---|---|---|---|---|---|---|
| | Suicide death rate per 100,000 (95% uncertainty interval) | | Percent change (95% uncertainty interval) | Suicide death rate per 100,000 (95% uncertainty interval) | | Percent change (95% uncertainty interval) |
| | 1990 | 2016 | 1990-2016 | 1990 | 2016 | 1990-2016 |
| **Overall** | **19.4 (16.1 to 22.9)** | **14.9 (13.2 to 16.4)** | **-23.5 (-38.2 to -3.2)** | **18.6 (12.1 to 22.8)** | **19.9 (13.8 to 22.2)** | **6.9 (-15.8 to 28.9)** |
| 10-14 | 4.8 (3.2 to 6.5) | 2.5 (1.9 to 3.2) | -47.1 (-65.5 to -13.4) | 2.6 (1.6 to 3.8) | 1.7 (1.3 to 2.2) | -34.6 (-56.6 to 5.2) |
| 15-19 | 43.8 (34.4 to 52.5) | 26.7 (22.9 to 30.8) | -39.0 (-52.5 to -16.9) | 16.5 (11.1 to 20.7) | 12.8 (9.9 to 15.1) | -22.3 (-42.1 to 3.0) |
| 20-24 | 51.5 (42.9 to 60.6) | 33.1 (28.5 to 37.6) | -35.8 (-49.4 to -17.2) | 32.6 (21.8 to 40.2) | 27.8 (20.2 to 31.8) | -14.7 (-32.8 to 5.2) |
| 25-29 | 40.9 (33.5 to 48.6) | 29.0 (25.1 to 32.7) | -29.1 (-45.0 to -8.2) | 36.2 (25 to 44) | 33.1 (24.6 to 37.5) | -8.6 (-27.1 to 10.5) |
| 30-34 | 26.0 (20.4 to 31.8) | 18.8 (16.2 to 21.5) | -27.4 (-44.8 to -1.8) | 34.4 (21.9 to 42.2) | 31.7 (22.6 to 36) | -7.9 (-27.9 to 13.7) |
| 35-39 | 21.4 (16.8 to 26.4) | 16.4 (14.2 to 18.7) | -23.5 (-41.4 to 2.5) | 32.5 (20.3 to 40.2) | 32.0 (21.1 to 36.4) | -1.4 (-23.7 to 23.0) |
| 40-44 | 17.0 (13.1 to 20.8) | 12.7 (11.1 to 14.5) | -25.3 (-42.1 to 2.6) | 29.2 (17.9 to 36.6) | 28.3 (18.3 to 32.4) | -3.2 (-26.3 to 20.4) |
| 45-49 | 14.8 (11.6 to 18.2) | 11.3 (10 to 13.1) | -23.8 (-41.1 to 1.8) | 29.4 (17 to 37.4) | 28.0 (17.6 to 32.1) | -4.9 (-27.7 to 19.6) |
| 50-54 | 15.5 (11.8 to 19.4) | 12.5 (11.1 to 14.2) | -19.5 (-35.5 to 7.3) | 27.6 (15.9 to 35.3) | 26.0 (16.2 to 29.9) | -5.8 (-28.6 to 19.7) |
| 55-59 | 12.6 (9.7 to 15.9) | 10.1 (8.8 to 11.7) | -19.9 (-37.7 to 9.5) | 30.2 (17.4 to 38.9) | 28.3 (17.9 to 32.2) | -6.2 (-29.5 to 20.3) |
| 60-64 | 13.7 (10.6 to 17.4) | 12.0 (10.4 to 14.1) | -12.7 (-31.7 to 16.4) | 25.5 (15.4 to 32.9) | 24.5 (16.1 to 28.3) | -4.1 (-28.6 to 22.8) |
| 65-69 | 15.8 (12.1 to 20.1) | 15.1 (12.9 to 17.6) | -4.7 (-26.3 to 29.6) | 26.9 (15.6 to 34.8) | 26.8 (17.2 to 31.2) | -0.6 (-25.4 to 28.4) |
| 70-74 | 15.5 (11.5 to 20) | 16.4 (13.7 to 19.1) | 5.7 (-18.2 to 43.2) | 25.6 (16 to 32.6) | 27.4 (17.8 to 32.1) | 7.0 (-20.6 to 40.1) |
| 75-79 | 18.0 (13.1 to 23.5) | 20.9 (17.5 to 24.4) | 16.0 (-11.0 to 58.0) | 33.4 (20.7 to 43.2) | 37.0 (24.9 to 43.3) | 10.9 (-16.9 to 42.9) |
| 80-84 | 17.1 (12.5 to 23) | 24.6 (19.9 to 28.8) | 44.0 (9.5 to 86.9) | 41.0 (27.6 to 53.9) | 55.0 (37.5 to 64.4) | 34.0 (1.8 to 82.8) |
| 85-89 | 17.9 (13.5 to 23.3) | 28.0 (22.8 to 32.3) | 56.3 (24.9 to 92.5) | 48.3 (33.7 to 62.7) | 67.5 (48.6 to 78.2) | 39.6 (6.1 to 90.6) |
| 90-94 | 19.2 (14.7 to 24.8) | 31.7 (26.4 to 36.3) | 65.3 (32.7 to 104.4) | 53.0 (37.2 to 68.2) | 77.4 (57.7 to 90.6) | 46.1 (9.3 to 100.4) |
| 95 plus | 23.9 (18.2 to 31.3) | 40.6 (32.6 to 47.6) | 69.7 (34.8 to 114.0) | 54.0 (37.9 to 74.8) | 80.8 (64 to 96.7) | 49.7 (9.2 to 111.4) |

## 7. Number and percent of suicide deaths by age group in India, 1990 and 2016

| Age group (years) | Women | | | | Men | | | |
|---|---|---|---|---|---|---|---|---|
| | Number of suicide deaths | | Percent of total suicide deaths | | Number of suicide deaths | | Percent of total suicide deaths | |
| | 1990 | 2016 | 1990 | 2016 | 1990 | 2016 | 1990 | 2016 |
| 10-14 | 2,246 | 1,511 | 2.77 | 1.60 | 1,342 | 1,160 | 1.61 | 0.85 |
| 15-19 | 18,494 | 15,685 | 22.82 | 16.62 | 7,540 | 8,394 | 9.04 | 6.18 |
| 20-24 | 19,329 | 18,681 | 23.85 | 19.79 | 13,291 | 17,403 | 15.94 | 12.80 |
| 25-29 | 13,677 | 15,808 | 16.88 | 16.75 | 13,268 | 19,636 | 15.92 | 14.45 |
| 30-34 | 7,646 | 9,623 | 9.43 | 10.20 | 11,180 | 17,440 | 13.41 | 12.83 |
| 35-39 | 5,518 | 7,411 | 6.81 | 7.85 | 9,272 | 15,486 | 11.12 | 11.39 |
| 40-44 | 3,432 | 5,088 | 4.23 | 5.39 | 6,284 | 12,050 | 7.54 | 8.86 |
| 45-49 | 2,561 | 3,995 | 3.16 | 4.23 | 5,331 | 10,424 | 6.39 | 7.67 |
| 50-54 | 2,407 | 3,877 | 2.97 | 4.11 | 4,369 | 8,434 | 5.24 | 6.20 |
| 55-59 | 1,621 | 2,683 | 2.00 | 2.84 | 3,954 | 7,755 | 4.74 | 5.71 |
| 60-64 | 1,353 | 2,634 | 1.67 | 2.79 | 2,627 | 5,472 | 3.15 | 4.03 |
| 65-69 | 1,125 | 2,348 | 1.39 | 2.49 | 1,975 | 4,013 | 2.37 | 2.95 |
| 70-74 | 741 | 1,764 | 0.91 | 1.87 | 1,218 | 2,697 | 1.46 | 1.98 |
| 75-79 | 505 | 1,523 | 0.62 | 1.61 | 898 | 2,322 | 1.08 | 1.71 |
| 80-84 | 260 | 1,038 | 0.32 | 1.10 | 521 | 1,935 | 0.62 | 1.42 |
| 85-89 | 97 | 509 | 0.12 | 0.54 | 228 | 960 | 0.27 | 0.71 |
| 90-94 | 24 | 164 | 0.03 | 0.17 | 59 | 300 | 0.07 | 0.22 |
| 95 plus | 5 | 38 | 0.01 | 0.04 | 9 | 52 | 0.01 | 0.04 |

## 8. Percent of total deaths due to suicide in young adults by sex in the states of India, 2016

| States of India* | Both sexes combined | | | | Women | | | | Men | | | |
|---|---|---|---|---|---|---|---|---|---|---|---|---|
| | Age group (years) | | | | Age group (years) | | | | Age group (years) | | | |
| | 15-29 | | 15-39 | | 15-29 | | 15-39 | | 15-29 | | 15-39 | |
| | Percent of total deaths due to suicide (95% uncertainty interval) | Rank of suicide deaths among all individual causes of death | Percent of total deaths due to suicide (95% uncertainty interval) | Rank of suicide deaths among all individual causes of death | Percent of total deaths due to suicide (95% uncertainty interval) | Rank of suicide deaths among all individual causes of death | Percent of total deaths due to suicide (95% uncertainty interval) | Rank of suicide deaths among all individual causes of death | Percent of total deaths due to suicide (95% uncertainty interval) | Rank of suicide deaths among all individual causes of death | Percent of total deaths due to suicide (95% uncertainty interval) | Rank of suicide deaths among all individual causes of death |
| Bihar | 7.0 (5.7 to 9.7) | 3 | 5.6 (4.7 to 7.7) | 6 | 7.0 (5.4 to 9.0) | 3 | 5.4 (4.3 to 6.5) | 5 | 7.0 (5.2 to 11.8) | 3 | 5.7 (4.5 to 9.3) | 6 |
| Jharkhand | 9.1 (7.1 to 12.4) | 3 | 7.0 (5.6 to 9.7) | 4 | 10.4 (7.4 to 13.6) | 2 | 7.6 (5.5 to 9.9) | 3 | 8.0 (5.8 to 13.1) | 3 | 6.4 (5.0 to 10.7) | 4 |
| Uttar Pradesh | 14.0 (11.9 to 16.3) | 1 | 10.8 (9.4 to 12.3) | 3 | 16.7 (13.3 to 20.8) | 1 | 12.5 (10.3 to 15.2) | 1 | 11.7 (9.0 to 14.3) | 2 | 9.5 (7.4 to 11.2) | 3 |
| Rajasthan | 11.5 (9.8 to 13.5) | 2 | 9.2 (8.0 to 10.8) | 2 | 12.5 (10.0 to 15.3) | 1 | 9.6 (7.9 to 11.4) | 1 | 10.8 (8.6 to 13.8) | 2 | 8.9 (7.4 to 11.2) | 3 |
| Meghalaya | 8.9 (7.4 to 11.6) | 2 | 7.2 (6.2 to 9.6) | 2 | 6.1 (4.7 to 7.9) | 5 | 4.7 (3.7 to 5.9) | 5 | 10.9 (8.7 to 15.5) | 1 | 8.7 (7.2 to 12.5) | 3 |
| Assam | 13.4 (11.1 to 16.0) | 1 | 10.1 (8.5 to 11.8) | 1 | 15.6 (11.9 to 20.0) | 1 | 11.3 (8.8 to 14.5) | 1 | 11.4 (9.0 to 15.0) | 2 | 9.1 (7.5 to 11.9) | 2 |
| Chhattisgarh | 16.0 (13.3 to 18.6) | 1 | 12.8 (10.3 to 14.7) | 1 | 15.3 (11.8 to 19.2) | 1 | 11.3 (8.7 to 14.1) | 1 | 16.5 (11.3 to 20.0) | 1 | 13.7 (9.1 to 16.4) | 1 |
| Madhya Pradesh | 16.4 (13.9 to 18.9) | 1 | 12.7 (10.9 to 14.5) | 1 | 18.3 (14.4 to 22.6) | 1 | 13.9 (11.0 to 17.1) | 1 | 15.0 (11.4 to 17.9) | 2 | 12.0 (8.9 to 14.1) | 2 |
| Odisha | 14.0 (11.7 to 16.9) | 1 | 10.3 (8.9 to 12.3) | 1 | 16.6 (13.0 to 20.9) | 1 | 11.9 (9.4 to 14.7) | 1 | 11.6 (9.1 to 15.0) | 2 | 9.2 (7.6 to 11.9) | 2 |
| Arunachal Pradesh | 17.6 (15.0 to 20.4) | 1 | 13.4 (11.5 to 15.4) | 1 | 22.1 (17.5 to 28.2) | 1 | 16.7 (13.3 to 21.2) | 1 | 14.3 (11.3 to 17.0) | 2 | 11.5 (9.0 to 13.5) | 1 |
| Mizoram | 6.7 (5.4 to 9.5) | 4 | 5.5 (4.5 to 8.0) | 4 | 4.8 (3.6 to 6.2) | 6 | 3.5 (2.7 to 4.5) | 7 | 8.1 (6.3 to 12.5) | 2 | 6.6 (5.3 to 10.5) | 3 |
| Nagaland | 5.7 (4.3 to 11.6) | 4 | 4.5 (3.4 to 9.4) | 4 | 5.3 (3.9 to 7.1) | 5 | 4.0 (2.9 to 5.2) | 7 | 6.0 (4.1 to 15.4) | 3 | 4.7 (3.3 to 12.1) | 4 |
| Uttarakhand | 12.9 (10.9 to 15.5) | 2 | 9.8 (8.5 to 12.1) | 2 | 17.7 (13.8 to 22.1) | 1 | 13.7 (11.0 to 16.9) | 1 | 9.6 (7.4 to 12.7) | 2 | 7.6 (6.1 to 10.3) | 3 |
| Gujarat | 18.4 (16.3 to 20.6) | 1 | 13.4 (12.0 to 14.8) | 1 | 25.3 (21.8 to 29.3) | 1 | 18.6 (16.2 to 21.1) | 1 | 13.6 (11.1 to 16.2) | 2 | 10.5 (8.6 to 12.1) | 3 |
| Tripura | 28.6 (19.7 to 33.3) | 1 | 22.7 (14.6 to 26.4) | 1 | 30.4 (24.8 to 36.3) | 1 | 23.5 (19.4 to 27.8) | 1 | 27.2 (12.2 to 33.2) | 1 | 22.2 (9.4 to 26.7) | 1 |
| Sikkim | 16.0 (13.7 to 19.6) | 1 | 12.4 (10.8 to 15.0) | 1 | 18.5 (14.7 to 24.1) | 1 | 14.2 (11.5 to 18.1) | 1 | 14.4 (11.5 to 19.3) | 2 | 11.5 (9.4 to 15.6) | 2 |
| Manipur | 13.0 (10.9 to 15.3) | 2 | 9.8 (8.3 to 11.3) | 3 | 14.0 (10.9 to 17.6) | 1 | 10.3 (8.1 to 12.8) | 1 | 12.2 (9.6 to 15.0) | 2 | 9.4 (7.5 to 11.3) | 3 |
| Haryana | 13.5 (11.4 to 15.3) | 2 | 10.4 (9.1 to 11.7) | 3 | 15.3 (12.6 to 19.0) | 1 | 11.6 (9.7 to 13.9) | 1 | 12.5 (10.0 to 14.9) | 2 | 9.9 (7.9 to 11.4) | 3 |
| Delhi | 10.4 (8.6 to 13.8) | 2 | 7.8 (6.5 to 10.7) | 3 | 12.1 (9.4 to 15.9) | 1 | 8.7 (7.0 to 11.0) | 2 | 9.3 (7.0 to 14.5) | 2 | 7.3 (5.7 to 11.6) | 4 |
| Telangana | 24.9 (20.5 to 29.2) | 1 | 18.6 (14.8 to 21.5) | 1 | 30.8 (25.8 to 37.0) | 1 | 22.9 (19.4 to 26.9) | 1 | 20.0 (11.7 to 24.9) | 1 | 15.8 (8.7 to 19.4) | 1 |
| Andhra Pradesh | 24.2 (19.4 to 27.9) | 1 | 18.1 (13.8 to 20.6) | 1 | 30.3 (25.7 to 36.0) | 1 | 22.4 (19.4 to 25.9) | 1 | 19.6 (11.1 to 23.6) | 1 | 15.4 (8.4 to 18.3) | 1 |
| Jammu and Kashmir | 10.4 (8.4 to 14.1) | 2 | 7.6 (6.3 to 10.5) | 3 | 15.4 (12.0 to 20.2) | 1 | 11.2 (8.8 to 14.5) | 1 | 6.8 (5.1 to 13.1) | 2 | 5.5 (4.3 to 10.5) | 3 |
| Karnataka | 27.1 (21.2 to 31.4) | 1 | 21.1 (15.5 to 24.1) | 1 | 32.9 (28.3 to 39.1) | 1 | 25.0 (21.4 to 29.4) | 1 | 22.7 (12.3 to 27.5) | 1 | 18.6 (9.2 to 22.2) | 1 |
| West Bengal | 24.6 (19.9 to 29.0) | 1 | 18.3 (14.1 to 21.1) | 1 | 31.0 (25.7 to 37.1) | 1 | 22.9 (19.2 to 26.6) | 1 | 19.3 (10.2 to 24.0) | 1 | 15.1 (7.9 to 18.1) | 1 |
| Maharashtra | 18.7 (16.3 to 21.3) | 1 | 14.3 (12.3 to 16.1) | 1 | 22.1 (18.4 to 26.2) | 1 | 17.0 (14.3 to 19.8) | 1 | 16.4 (12.2 to 19.6) | 2 | 12.9 (9.3 to 15.0) | 2 |
| UTs other than Delhi† | 21.9 (18.7 to 25.1) | 1 | 16.5 (13.8 to 18.8) | 1 | 31.6 (26.7 to 37.6) | 1 | 23.5 (20.0 to 27.6) | 1 | 16.7 (11.6 to 20.2) | 2 | 13.5 (8.9 to 16.1) | 1 |
| Himachal Pradesh | 17.4 (14.8 to 20.5) | 1 | 13.2 (11.5 to 15.6) | 2 | 21.9 (17.3 to 27.4) | 1 | 16.0 (12.6 to 20.0) | 1 | 14.7 (11.8 to 18.7) | 2 | 11.9 (9.7 to 14.8) | 2 |
| Punjab | 9.8 (8.2 to 13.3) | 3 | 7.3 (6.2 to 10.1) | 3 | 12.8 (10.2 to 15.8) | 1 | 9.2 (7.5 to 11.2) | 2 | 7.8 (6.0 to 13.2) | 3 | 6.2 (4.9 to 10.4) | 3 |
| Goa | 18.7 (16.5 to 21.6) | 1 | 13.0 (11.4 to 15.0) | 1 | 30.4 (25.7 to 35.8) | 1 | 21.1 (18.1 to 24.7) | 1 | 12.6 (10.3 to 15.6) | 2 | 9.4 (7.9 to 11.7) | 3 |
| Tamil Nadu | 31.1 (24.6 to 35.8) | 1 | 23.0 (17.2 to 26.3) | 1 | 42.9 (37.9 to 49.2) | 1 | 32.1 (28.3 to 36.5) | 1 | 22.7 (11.7 to 27.7) | 1 | 17.7 (8.9 to 21.2) | 1 |
| Kerala | 29.0 (22.0 to 33.1) | 1 | 22.7 (15.4 to 25.8) | 1 | 35.3 (30.5 to 41.1) | 1 | 25.3 (21.9 to 29.4) | 1 | 24.9 (11.9 to 30.1) | 1 | 21.3 (8.9 to 25.7) | 1 |

*The states are listed in increasing order of epidemiological transition level in 2016. †Union territories.

**9. Men-to-women suicide crude death rate ratio by age in India and in the states grouped by epidemiological transition level, 1990 and 2016**

| Age group (years) | India | | ETL state group | | | | | | | |
|---|---|---|---|---|---|---|---|---|---|---|
| | | | Low | | Lower-middle | | Higher-middle | | High | |
| | 1990 | 2016 | 1990 | 2016 | 1990 | 2016 | 1990 | 2016 | 1990 | 2016 |
| **Overall** | **0.96** | **1.34** | **0.96** | **1.35** | **0.91** | **1.18** | **0.97** | **1.34** | **0.97** | **1.41** |
| 10-14 | 0.56 | 0.69 | 0.62 | 0.74 | 0.47 | 0.62 | 0.55 | 0.68 | 0.52 | 0.58 |
| 15-19 | 0.38 | 0.48 | 0.41 | 0.50 | 0.35 | 0.44 | 0.38 | 0.48 | 0.36 | 0.44 |
| 20-24 | 0.63 | 0.84 | 0.65 | 0.89 | 0.64 | 0.76 | 0.63 | 0.84 | 0.61 | 0.77 |
| 25-29 | 0.89 | 1.14 | 0.91 | 1.21 | 0.90 | 1.06 | 0.88 | 1.12 | 0.89 | 1.08 |
| 30-34 | 1.32 | 1.68 | 1.41 | 1.86 | 1.38 | 1.51 | 1.29 | 1.59 | 1.22 | 1.58 |
| 35-39 | 1.52 | 1.95 | 1.57 | 2.10 | 1.61 | 1.94 | 1.54 | 1.90 | 1.42 | 1.86 |
| 40-44 | 1.72 | 2.23 | 1.56 | 2.22 | 1.84 | 2.15 | 1.79 | 2.25 | 1.78 | 2.27 |
| 45-49 | 1.98 | 2.48 | 1.84 | 2.55 | 1.84 | 2.06 | 2.00 | 2.45 | 2.17 | 2.63 |
| 50-54 | 1.78 | 2.08 | 1.78 | 2.26 | 1.71 | 1.68 | 1.79 | 2.01 | 1.93 | 2.22 |
| 55-59 | 2.39 | 2.80 | 2.34 | 2.99 | 2.23 | 2.20 | 2.28 | 2.64 | 2.63 | 2.99 |
| 60-64 | 1.86 | 2.04 | 1.89 | 2.25 | 1.81 | 1.63 | 1.86 | 1.96 | 1.93 | 2.15 |
| 65-69 | 1.70 | 1.77 | 1.64 | 1.86 | 1.59 | 1.50 | 1.64 | 1.76 | 1.94 | 1.83 |
| 70-74 | 1.65 | 1.67 | 1.58 | 1.75 | 1.65 | 1.46 | 1.68 | 1.67 | 1.80 | 1.74 |
| 75-79 | 1.85 | 1.77 | 1.78 | 1.89 | 1.83 | 1.61 | 1.77 | 1.71 | 2.13 | 1.89 |
| 80-84 | 2.40 | 2.23 | 2.39 | 2.44 | 2.41 | 2.08 | 2.36 | 2.18 | 2.82 | 2.31 |
| 85-89 | 2.70 | 2.41 | 2.70 | 2.64 | 2.60 | 2.18 | 2.68 | 2.36 | 3.16 | 2.57 |
| 90-94 | 2.76 | 2.44 | 2.82 | 2.69 | 2.59 | 2.19 | 2.77 | 2.41 | 3.20 | 2.71 |
| 95 plus | 2.26 | 1.99 | 2.31 | 2.15 | 2.10 | 1.78 | 2.28 | 1.98 | 2.62 | 2.33 |

ETL is epidemiological transition level.

## 10. Men-to-women suicide death rate ratio in the states of India, 1990 to 2016

| States of India | Men-to-women suicide mortality rate ratio | | | | | |
|---|---|---|---|---|---|---|
| | 1990 | 1995 | 2000 | 2005 | 2010 | 2016 |
| **India** | **0.96** | **0.91** | **0.99** | **1.05** | **1.27** | **1.34** |
| **Low ETL group** | **0.96** | **0.91** | **0.97** | **1.02** | **1.29** | **1.35** |
| Bihar | 1.00 | 0.96 | 1.01 | 1.00 | 1.29 | 1.36 |
| Jharkhand | 0.92 | 0.90 | 1.00 | 1.07 | 1.26 | 1.22 |
| Uttar Pradesh | 0.85 | 0.80 | 0.84 | 0.88 | 1.1 | 1.10 |
| Rajasthan | 1.50 | 1.47 | 1.57 | 1.56 | 1.68 | 1.83 |
| Meghalaya | 2.69 | 2.56 | 2.76 | 2.93 | 3.48 | 3.59 |
| Assam | 0.89 | 0.78 | 0.84 | 0.91 | 1.23 | 1.35 |
| Chhattisgarh | 1.58 | 1.58 | 1.73 | 1.83 | 2.16 | 2.34 |
| Madhya Pradesh | 0.97 | 0.93 | 0.98 | 1.04 | 1.40 | 1.59 |
| Odisha | 0.84 | 0.76 | 0.85 | 0.92 | 1.15 | 1.27 |
| **Lower-middle ETL group** | **0.91** | **0.88** | **0.97** | **0.96** | **1.12** | **1.18** |
| Arunachal Pradesh | 0.90 | 0.84 | 0.89 | 0.95 | 1.16 | 1.35 |
| Mizoram | 2.45 | 2.56 | 2.83 | 3.01 | 3.74 | 4.11 |
| Nagaland | 1.25 | 1.31 | 1.51 | 1.79 | 2.30 | 2.46 |
| Uttarakhand | 0.74 | 0.74 | 0.82 | 0.88 | 1.00 | 1.15 |
| Gujarat | 0.90 | 0.86 | 0.94 | 0.91 | 1.05 | 1.07 |
| Tripura | 1.03 | 1.06 | 1.24 | 1.36 | 1.73 | 1.80 |
| Sikkim | 0.89 | 0.94 | 1.09 | 1.23 | 1.59 | 1.85 |
| Manipur | 1.52 | 1.14 | 1.60 | 1.57 | 1.74 | 1.89 |
| **Higher-middle ETL group** | **0.97** | **0.94** | **1.04** | **1.12** | **1.29** | **1.34** |
| Haryana | 1.10 | 1.13 | 1.46 | 1.72 | 1.89 | 1.89 |
| Delhi | 1.12 | 1.16 | 1.24 | 1.24 | 1.59 | 1.61 |
| Telangana | 0.84 | 0.80 | 0.89 | 0.94 | 1.13 | 1.25 |
| Andhra Pradesh | 0.92 | 0.85 | 0.93 | 0.99 | 1.21 | 1.37 |
| Jammu and Kashmir | 0.69 | 0.68 | 0.75 | 0.80 | 0.90 | 0.97 |
| Karnataka | 1.23 | 1.15 | 1.28 | 1.31 | 1.45 | 1.44 |
| West Bengal | 0.82 | 0.82 | 0.90 | 1.00 | 1.08 | 1.13 |
| Maharashtra | 1.12 | 1.14 | 1.17 | 1.29 | 1.52 | 1.52 |
| UTs other than Delhi* | 0.87 | 0.87 | 0.95 | 0.99 | 1.16 | 1.27 |
| **High ETL group** | **0.97** | **0.89** | **0.96** | **1.03** | **1.28** | **1.41** |
| Himachal Pradesh | 0.87 | 0.86 | 1.05 | 1.31 | 1.70 | 1.84 |
| Punjab | 1.02 | 1.03 | 1.13 | 1.20 | 1.42 | 1.44 |
| Tamil Nadu | 0.82 | 0.72 | 0.77 | 0.84 | 1.09 | 1.22 |
| Goa | 0.77 | 0.83 | 0.92 | 0.94 | 1.09 | 1.12 |
| Kerala | 1.77 | 1.77 | 1.06 | 2.00 | 2.25 | 2.23 |

ETL is epidemiological transition level. *Union territories.

**11. Projected suicide death rate for both sexes combined and the probability of reaching the SDG 2030 target for the states of India**

| States of India* | Age-standardised suicide death rate per 100,000 in 2015 | SDG target for suicide death rate per 100,000 in 2030† | Projected age-standardised suicide death rate per 100,000 in 2030 (95% uncertainty interval) | Percent probability of reaching the SDG 2030 target |
|---|---|---|---|---|
| **India** | **18.2** | **12.1** | **15.7 (13.0 to 19.1)** | **0** |
| Bihar | 9.0 | 6.0 | 9.4 (6.6 to 12.8) | 0.1 |
| Jharkhand | 10.2 | 6.8 | 8.4 (5.3 to 13.7) | 10.3 |
| Uttar Pradesh | 16.0 | 10.7 | 16.1 (11.4 to 22.0) | 0 |
| Rajasthan | 13.1 | 8.7 | 14.1 (9.8 to 19.8)) | 0 |
| Meghalaya | 9.0 | 6.0 | 8.6 (5.7 to 13.1) | 0.6 |
| Assam | 16.8 | 11.2 | 12.1 (8.1 to 18.1) | 36.7 |
| Chhattisgarh | 20.8 | 13.9 | 19.2 (12.9 to 27.0) | 0.2 |
| Madhya Pradesh | 18.7 | 12.5 | 16.9 (11.7 to 23.3) | 0.9 |
| Odisha | 15.4 | 10.3 | 11.3 (7.4 to 17.0) | 30.1 |
| Arunachal Pradesh | 18.0 | 12.0 | 14.3 (9.1 to 21.0) | 12.6 |
| Mizoram | 7.0 | 4.7 | 6.6 (4.3 to 10.7) | 0.4 |
| Nagaland | 5.2 | 3.5 | 5.0 (2.9 to 11.5) | 0.6 |
| Uttarakhand | 13.3 | 8.9 | 9.7 (6.5 to 14.6) | 27.8 |
| Gujarat | 16.7 | 11.1 | 14.1 (10.4 to 19.1) | 2.1 |
| Tripura | 30.1 | 20.1 | 26.9 (14.0 to 39.4) | 0.8 |
| Sikkim | 13.1 | 8.7 | 9.7 (6.2 to 15.4) | 29.9 |
| Manipur | 13.4 | 8.9 | 12.8 (8.6 to 18.8) | 0.6 |
| Haryana | 15.7 | 10.5 | 14.0 (9.7 to 19.1) | 0.3 |
| Delhi | 7.3 | 4.9 | 6.0 (3.9 to 9.4) | 7.8 |
| Telangana | 22.3 | 14.9 | 16.1 (9.9 to 24.7) | 34.6 |
| Andhra Pradesh | 24.7 | 16.5 | 20.3 (12.4 to 28.8) | 6.2 |
| Jammu and Kashmir | 8.9 | 5.9 | 7.0 (4.8 to 11.4) | 9.3 |
| Karnataka | 30.1 | 20.1 | 25.7 (16.0 to 35.8) | 2.3 |
| West Bengal | 23.0 | 15.3 | 18.1 (12.1 to 25.0) | 6.4 |
| Maharashtra | 18.4 | 12.3 | 16.8 (11.9 to 22.4) | 0.2 |
| UTs other than Delhi‡ | 16.8 | 11.2 | 12.9 (8.5 to 18.6) | 15 |
| Himachal Pradesh | 12.4 | 8.3 | 10.2 (7.1 to 14.4) | 5.6 |
| Punjab | 9.1 | 6.1 | 8.0 (5.4 to 12.1) | 1.1 |
| Tamil Nadu | 29.2 | 19.5 | 21.1 (13.2 to 29.5) | 25.1 |
| Goa | 11.0 | 7.3 | 9.0 (6.7 to 12.3) | 6.3 |
| Kerala | 19.9 | 13.3 | 17.0 (10.1 to 23.6) | 0.7 |

*The states are listed in increasing order of epidemiological transition level in 2016. †One-third reduction from 2015 to 2030. ‡Union territories. SDG is sustainable development goal.

**12. All-age suicide death rate among women and men in states grouped by epidemiological transition level by age, 1990 and 2016**

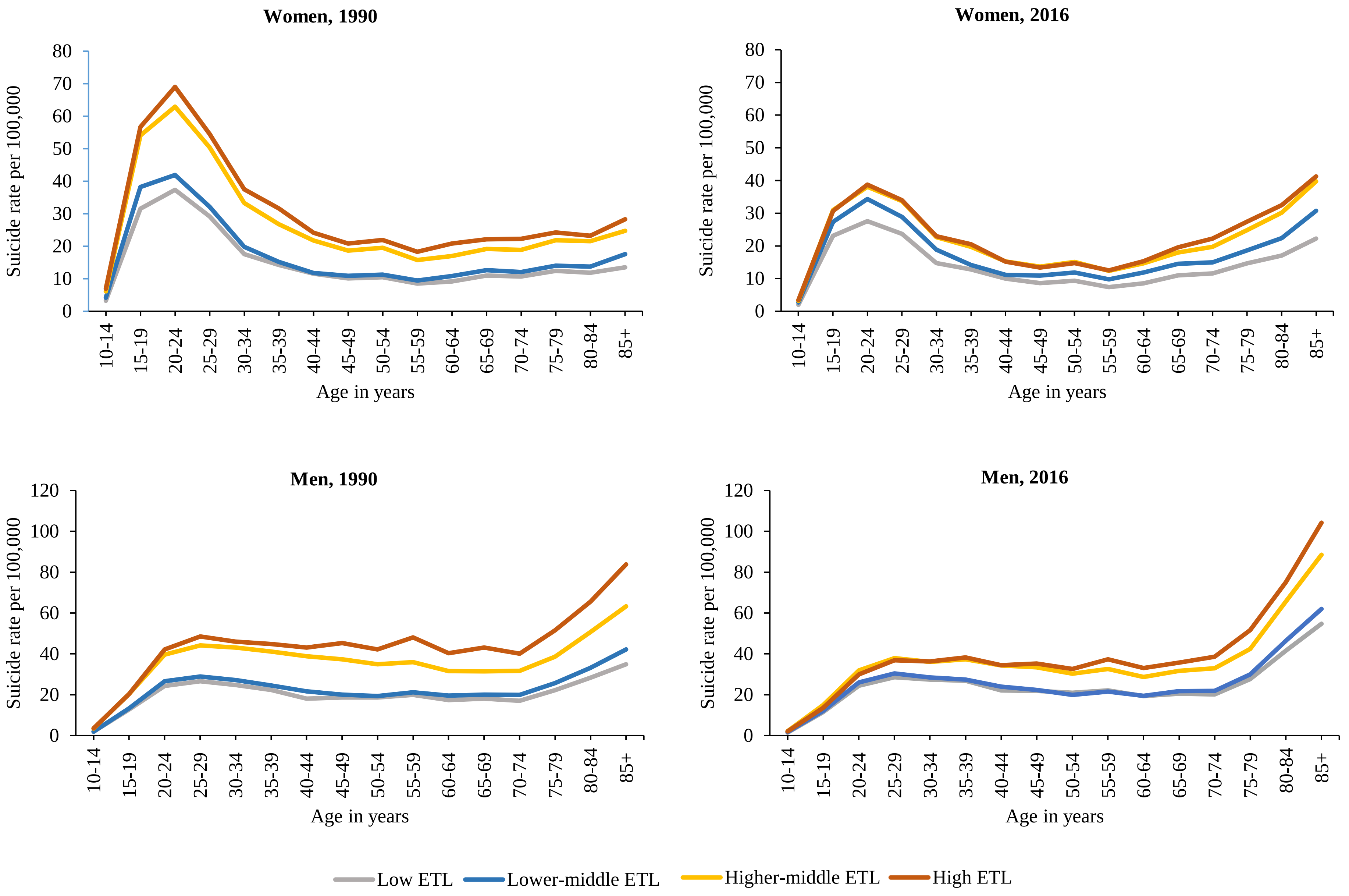


ETL is epidemiological transition level.
